# Modern Treatment of Neurogenic Thoracic Outlet Syndrome: Pathoanatomy, Diagnosis, and Arthroscopic Surgical Technique

**DOI:** 10.1016/j.jhsg.2022.07.004

**Published:** 2023-01-18

**Authors:** Adil S. Ahmed, Thibault Lafosse, Alexander R. Graf, Anthony L. Karzon, Michael B. Gottschalk, Eric R. Wagner

**Affiliations:** ∗ Department of Orthopaedic Surgery, Hand & Upper Extremity Surgery, Emory University School of Medicine, Atlanta, GA; † Upper Limb, Brachial Plexus, and Microsurgery, Alps Surgery Institute, Clinique Générale d’Annecy, Annecy, France

**Keywords:** Endoscopic brachial plexus neurolysis, Neurogenic thoracic outlet syndrome, Pectoralis minor release, Pectoralis minor syndrome, Suprascapular neuropathy

## Abstract

Compressive pathology in the supraclavicular and infraclavicular fossae is broadly termed “thoracic outlet syndrome,” with the large majority being neurogenic in nature. These are challenging conditions for patients and physicians and require robust knowledge of thoracic outlet anatomy and scapulothoracic kinematics to elucidate neurogenic versus vascular disorders. The combination of repetitive overhead activity and scapular dyskinesia leads to contracture of the scalene muscles, subclavius, and pectoralis minor, creating a chronically distalized and protracted scapular posture. This decreases the volume of the scalene triangle, costoclavicular space, and retropectoralis minor space, with resultant compression of the brachial plexus causing neurogenic thoracic outlet syndrome. This pathologic cascade leading to neurogenic thoracic outlet syndrome is termed pectoralis minor syndrome when primary symptoms localize to the infraclavicular area. Making the correct diagnosis is challenging and requires the combination of complete history, physical examination, advanced imaging, and ultrasound-guided injections. Most patients improve with nonsurgical treatment incorporating pectoralis minor stretching and periscapular and postural retraining. Surgical decompression of the thoracic outlet is reserved for compliant patients who fail nonsurgical management and respond favorably to targeted injections. In addition to prior exclusively open procedures with supraclavicular, infraclavicular, and/or transaxillary approaches, new minimally invasive and targeted endoscopic techniques have been developed over the past decade. They involve the endoscopic release of the pectoralis minor tendon, with additional suprascapular nerve release, brachial plexus neurolysis, and subclavius and interscalene release depending on the preoperative work-up.

Unlike other compressive neuropathies in the upper extremity, thoracic outlet syndrome (TOS) is less common and often more challenging to manage.^1^, ^2^, ^3^, ^4^ Broadly categorized as neurogenic TOS (NTOS) or vascular TOS (VTOS) (Table 1), approximately 90% to 95% of cases are neurogenic.^5^^,^^6^ The brachial plexus and accompanying subclavian vessels (Fig. 1) are subject to multiple potential sites of compression through the neck, supraclavicular and infraclavicular spaces, axilla, and upper arm.^5^ Traditionally, neurogenic symptoms were postulated to arise exclusively from brachial plexus compression between the anterior and middle scalenes or between the clavicle and first rib. This guided decades of treatment toward open first rib resection and scalenectomy for TOS in general, despite subpar results from this treatment in NTOS subgroups compared with those in VTOS subgroups.^7^, ^8^, ^9^, ^10^ However, contemporary understanding of the dynamic role of the pectoralis minor (PM) influencing scapular kinematics and infraclavicular compression led to the recognition of pectoralis minor syndrome (PMS) as one of the principal etiologies underlying NTOS.^11^ This supported an alternate approach to the surgical management of NTOS by targeting the PM for simultaneous decompression of the brachial plexus and correction of scapular dyskinesia.^6^^,^^8^^,^^12^

**Table 1:** NTOS Versus VTOS

	Neurogenic		Vascular
Distribution	90%–95%		5%–10% (venous >> arterial)
Demographic characteristics	Predominantly younger females		Predominantly younger, athletic males
Primary anatomic site	Retropectoralis minor space		Costoclavicular space, scalene triangle
Pathoanatomy	PM hyperactivity results in shortening and fibrosis Scapula assumes chronically protracted posture, decreasing volume of retropectoralis minor space Compression during arm elevation on brachial plexus cords +/− tethering of the suprascapular nerve at the suprascapular notch		Contraction of the anterior and middle scalene muscles superiorly elevates first rib relative to the clavicle, decreasing costoclavicular space Primarily compresses the subclavian vein and to lesser extent the subclavian artery Anatomic variations affecting costoclavicular space are common (cervical rib, anomalous scalene, enlarged transverse process, etc)
Primary symptoms	Pain around the shoulder, neck, trapezius, and medial scapula, often accompanied by muscle spasms Subjective paresthesias in the arm or hand may be present but are nonspecific		Hand and finger pain, cold intolerance, claudication, and episodic arm swelling Arm heaviness and easy fatiguability with use Subjective paresthesias in the arm or hand may be present but are nonspecific
Examination findings	Tenderness and positive Tinel sign over PM (and less commonly over scalenes) Scapular dyskinesia Hand atrophy—late presentation (Gilliatt-Sumner hand)		Unilateral arm swelling and cyanosis Venous distention around the upper arm Raynaud-type appearance and skin changes in the fingers
Traditional maneuvers	Multiple described provocative examinations (Adson, Wright, Roos, Cyriax, etc) are nonspecific, with high false-positive rates even in the normal population		
Measurements (compared with the contralateral side)	PM Index Medial scapular distance Medial scapular angle Scapular protraction height		
Diagnostic work-up	Ultrasound-guided anesthetic injections—target PM coracoid insertion with or without suprascapular nerve at suprascapular notch with or without scalene triangle Magnetic resonance angiogram of the chest—arms down/arms up protocol for dynamic vascular compression Magnetic resonance imaging of the brachial plexus EMG/NCS of bilateral upper extremities		
Surgical treatment	PM release (open or arthroscopic) With or without suprascapular nerve release With or without brachial plexus neurolysis With or without subclavius release	First rib resection (transaxillary or supraclavicular) With or without scalenectomy With or without resection of anomalous anatomy (if present)

**Figure 1:**
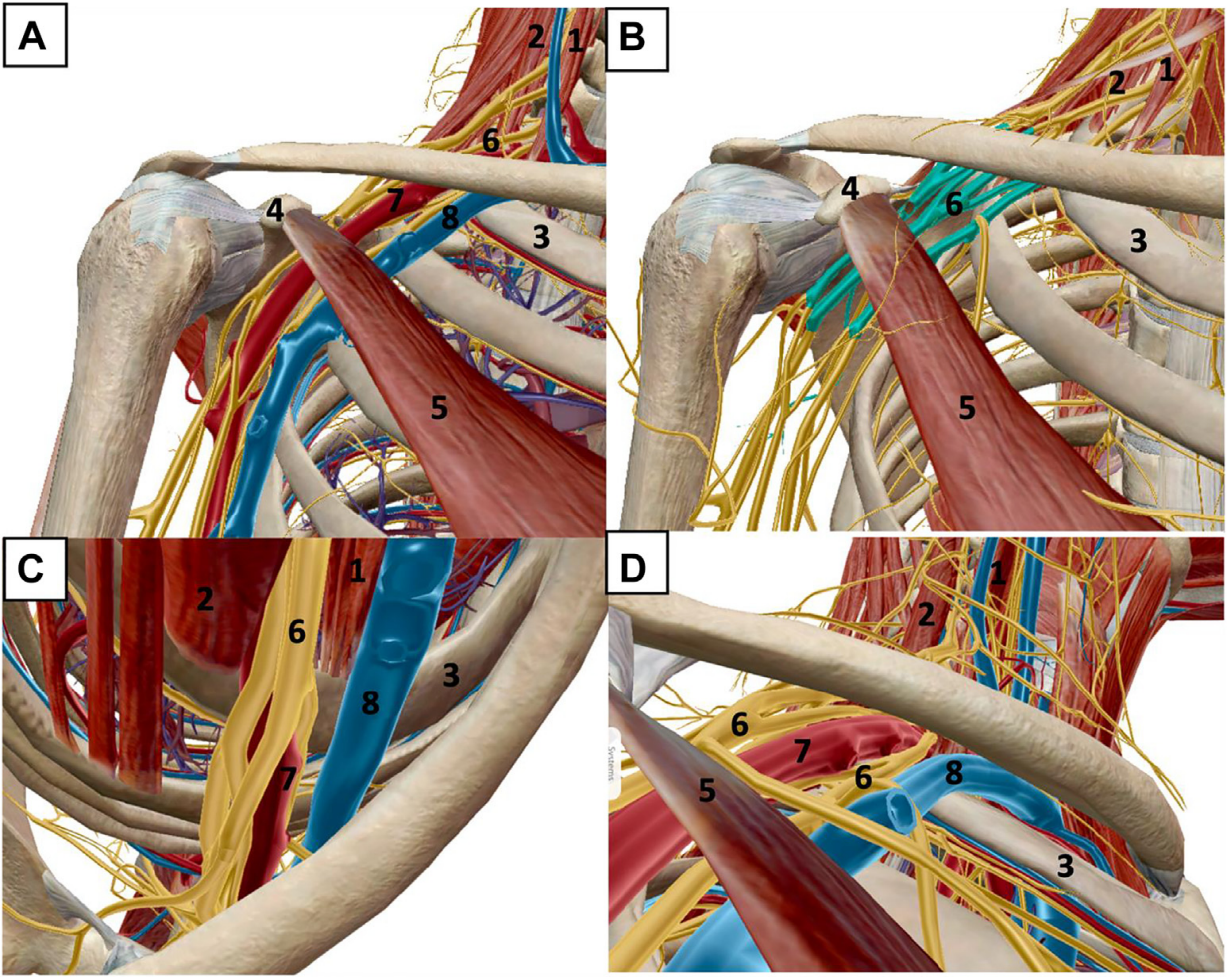
Rendering of the brachial plexus and subclavian/axillary vessels passing through the thoracic outlet in the right shoulder. **A** Anterior view with subtraction of the deltoid, pectoralis major, trapezius, rotator cuff, and conjoint tendon. **B** Brachial plexus highlighted at the cord level deep to the pectoralis minor, with subtraction of the vasculature. **C** Inlet view (looking from superior to inferior) of the thoracic outlet. The subclavian vein courses anterior to the anterior scalene in the costoclavicular space. The brachial plexus and subclavian artery pass between the anterior and middle scalenes. **D** Outlet view (looking from inferior to superior) of the thoracic outlet. (1) anterior scalene, (2) middle scalene, (3) first rib, (4) coracoid process, (5) pectoralis minor muscle, (6) brachial plexus, (7) subclavian artery, and (8) subclavian vein.

Neurogenic TOS presents diagnostic and treatment conundrums for several reasons. Thoracic outlet anatomy lies at the intersection between treating subspecialties with variable training regarding nerve surgery, open exploration, and less invasive endoscopic techniques.^13^, ^14^, ^15^, ^16^, ^17^ There is no consistent diagnostic algorithm; thus, patients with NTOS are often shuffled between different treating subspecialties, even undergoing several procedures before the correct diagnosis is made.^18^ Finally, patients’ psychological state often influences the perception of their pathology and ultimate outcome and should be assessed before invasive treatment. This review provides a comprehensive overview of NTOS, highlighting the anatomy, dynamic pathophysiology, reproducible diagnostic algorithm, and modern endoscopic management techniques for complete infraclavicular thoracic outlet decompression.

## Thoracic outlet anatomy and biomechanics

### The scalene triangle

**Figure 2:**
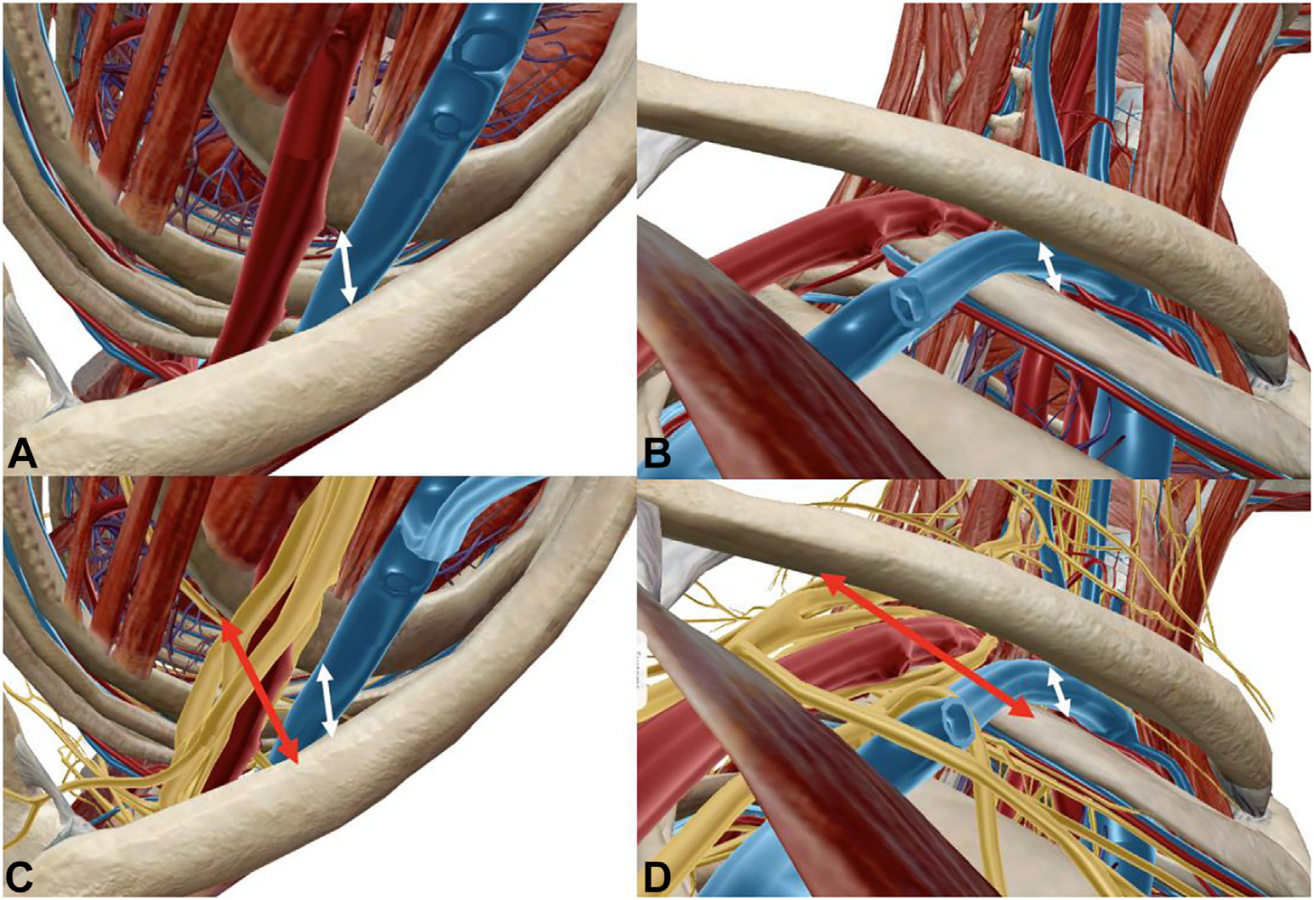
Inlet and outlet renderings illustrating anatomic relationships of the neurovascular bundle and osseous structures. **A** Inlet view with subtraction of the brachial plexus, demonstrating the proximity of the subclavian vessels between the clavicle and first rib. **B** Outlet view depicting a similar relationship between the vessels and osseous anatomy. **C** Inlet view with the brachial plexus. Note the relatively posterior position of the plexus and increased distance between the clavicle and first rib at this location. **D** Outlet view with the brachial plexus. Again, note the markedly increased distance between the clavicle and first rib at the posterior location of the plexus.

The thoracic outlet is broadly divided into supraclavicular and infraclavicular fossae (Fig. 1). The supraclavicular fossa contains 2 anatomic spaces: the scalene triangle and costoclavicular space.^19^^,^^20^ The subclavian artery courses inferiorly within the scalene triangle and anterior to the brachial plexus, in close proximity to the first rib, whereas the subclavian vein courses anterior to the anterior scalene (Fig. 2).^21^, ^22^, ^23^, ^24^ The scalenes elevate the first rib superiorly and tilt the neck to the ipsilateral side, as they originate from the transverse processes of the cervical vertebrae.^25^ First rib elevation decreases the volume of the scalene triangle.^26^ The subclavian artery is in closest proximity to the first rib, and it is the first structure in this space subject to compression during this dynamic process.^19^^,^^27^ Additionally, anterior scalene variations with split insertion can envelop and further compress the artery.^28^ The roots and trunks of the plexus, particularly the upper and middle trunk, are further proximal and posterior and less likely to be compromised (Fig. 2).^29^^,^^30^ Therefore, the anatomy of the scalene triangle and relative proximity of vessels to the sites of compression support the belief that scalene triangle pathology is more likely to create VTOS. Of note, the C8/T1 roots and inferior trunk are the closest components of the brachial plexus to the first rib and C7 transverse cervical process. Thus, they are the most likely neural elements to be compromised by variations within the scalene triangle (ie, accessory scalenes, enlarged transverse processes, fibrotic bands, suspensory pleural dome ligaments, and others).^31^, ^32^, ^33^

### The costoclavicular space

Beyond the scalene triangle, the neurovascular bundle enters the costoclavicular space. This is anterior and inferior relative to the scalene triangle. However, because of the curved anatomy of the thoracic wall and clavicle, the long axis of this space is superior anteromedial to inferior-posterolateral.^34^, ^35^, ^36^ First rib elevation compresses the subclavian vein (and, to a lesser extent, the subclavian artery) against the undersurface of the clavicle (Fig. 2).^34^^,^^37^ Simultaneous subclavius muscle contraction or hypertrophy exacerbates this phenomenon.^38^^,^^39^ Variant anatomy, such as cervical ribs or enlarged vertebral transverse processes, preferentially decrease the volume in the anterior aspect of the costoclavicular space, compressing the subclavian vessels.^40^^,^^41^ Given the aforementioned orientation and dimensions of the costoclavicular space, the brachial plexus is relatively posterior and further from sources of compression than the vessels (Fig. 2).

### The retropectoralis minor space

**Figure 3:**
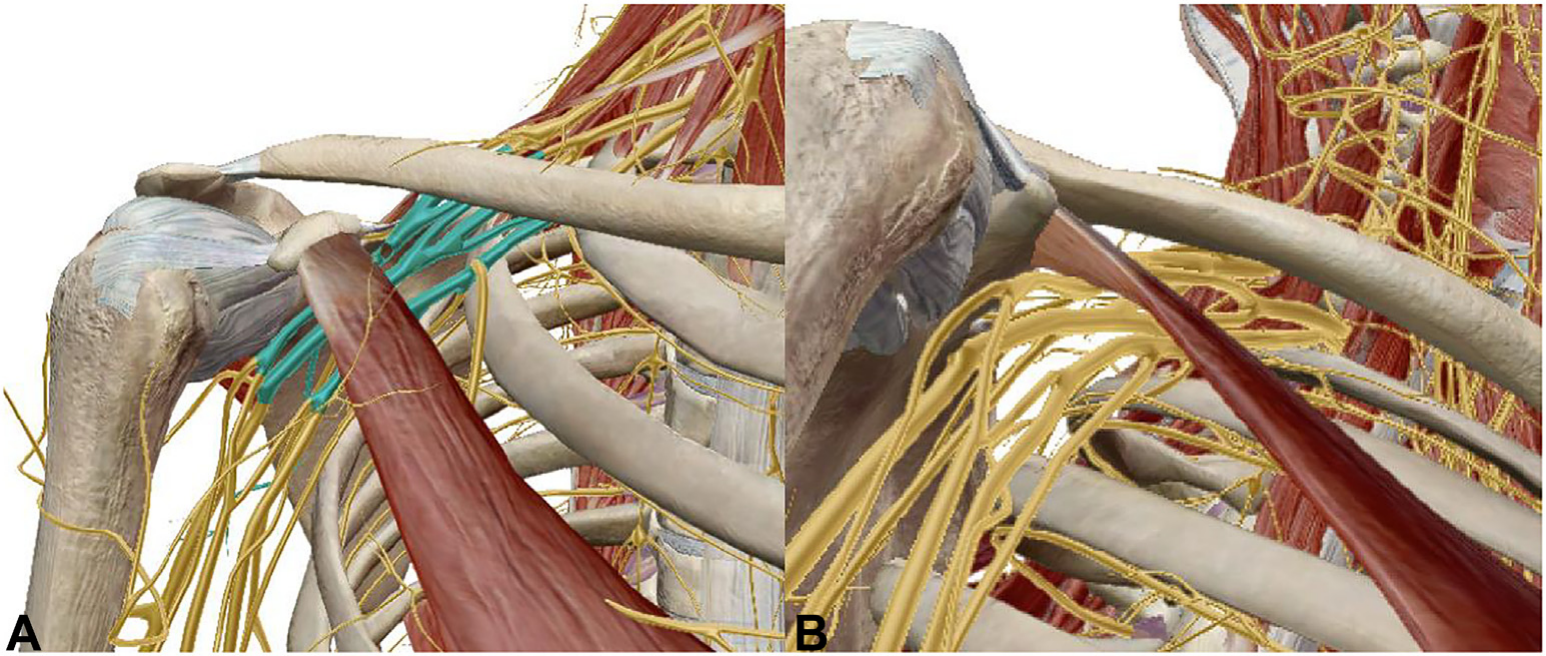
Images illustrating the relationship between the brachial plexus and PM within the retropectoralis minor space. **A** Anterior view, with the brachial plexus highlighted at the level of the cords. **B** Outlet view of the retropectoralis minor space, again demonstrating the proximity of the cords to the undersurface of the PM.

The prime space in the infraclavicular thoracic outlet is the retropectoralis minor space (Fig. 1), also called the subcoracoid space or thoracocoracopectoral space.^42^ It is bound by the coracoid superiorly, second through fourth ribs posteriorly, and PM anteriorly (Fig. 3).^12^^,^^43^ In this space, plexus divisions rejoin to form the lateral, medial, and posterior cords, and the second stage of the axillary artery continues deep to the PM.^44^ The PM is the principal dynamic driver controlling the retropectoralis minor space volume.^6^^,^^11^^,^^12^

### The PM and scapulothoracic abnormal motion

**Figure 4:**
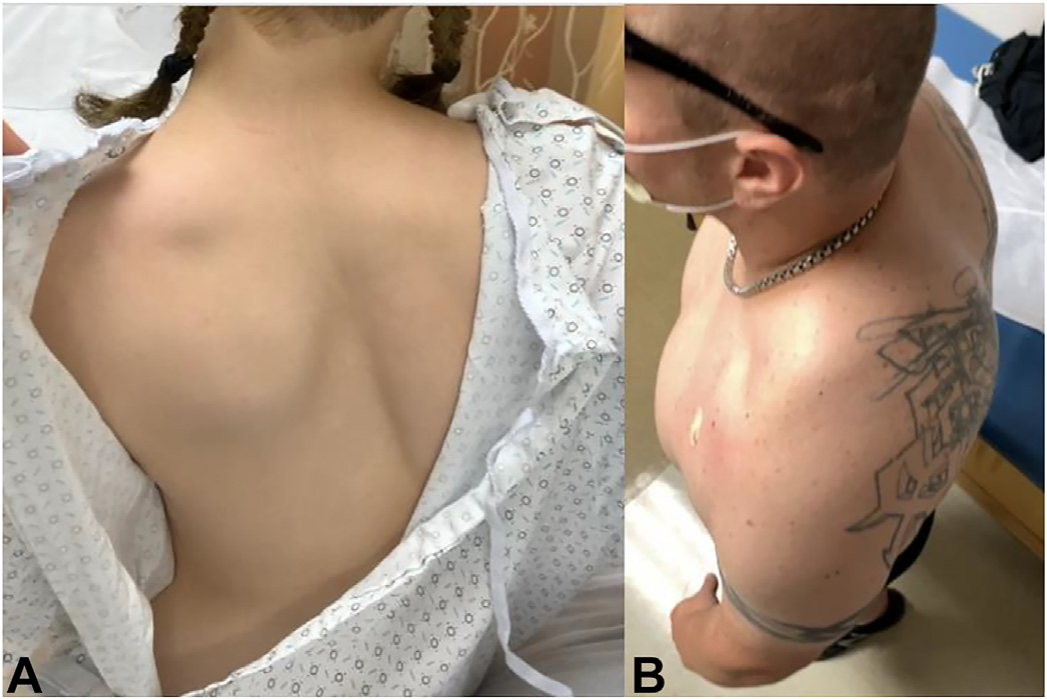
Appearance of the scapular protraction in 2 patients. **A** Posterior view with protraction of the left scapula, creating posterior elevation and prominence of the inferior angle. **B** Lateral and superior views demonstrating protracted resting position of the left scapula, with prominence of the inferior angle posteriorly.

The PM originates from costal cartilage margins of the third through fifth ribs and inserts onto the superomedial aspect of the coracoid, functioning as a dynamic scapular stabilizer (Fig. 3).^45^, ^46^, ^47^ The abnormalities of the PM alter scapular kinematics, particularly during repetitive movements with scapular protraction, as this position brings the coracoid insertion of the PM closer to its rib origin.^48^^,^^49^ Over time, a hyperactive or spasming PM shortens and develops contracture, leading to resting scapular protraction (Fig. 4) and altered scapular contribution to shoulder motion.^50^ Patients with a shortened PM exhibit scapular dyskinesia, more accurately referred to as scapulothoracic abnormal motion.^51^ It manifests as decreased scapular external rotation/retraction and posterior tilting of the inferior scapula compared with controls (Fig. 4).^52^

The loss of coordinated scapular rotation alters the normal harmony of glenohumeral to scapulothoracic motion, known as the scapulohumeral rhythm.^53^, ^54^, ^55^, ^56^ Aberrations in concerted humeral and scapular motion produce greater tuberosity impingement against the acromion during arm elevation.^57^ This pathologic cascade is especially prevalent and limiting for overhead athletes, who as a group maintain a more protracted scapular posture and shortened PM in their dominant arms.^58^, ^59^, ^60^, ^61^, ^62^ Burkhart et al^63^ coined “SICK” scapula syndrome as causing anterior shoulder pain in overhead athletes, describing the static anteriorly tilted coracoid malposition with tightening, shortening, and tenderness of the PM.^64^ This phenomenon is not limited to “overhead athletes” and is often encountered in manual workers participating in repetitive overhead activity.

Progressive PM tightness, shortening, and fibrosis, combined with anterior coracoid tilt, decreases the volume of the retropectoralis minor space.^42^ As the brachial plexus travels to the axilla and upper arm (Figure 1, Figure 3), the decreased volume leads to compression of the medial, lateral, and posterior cords and is especially pronounced during overhead activity. Tightness of the PM can exacerbate scapulothoracic abnormal motion, decreasing retropectoralis minor space volume and creating brachial plexus compression in NTOS.^65^

### Suprascapular neuropathy

**Figure 5:**
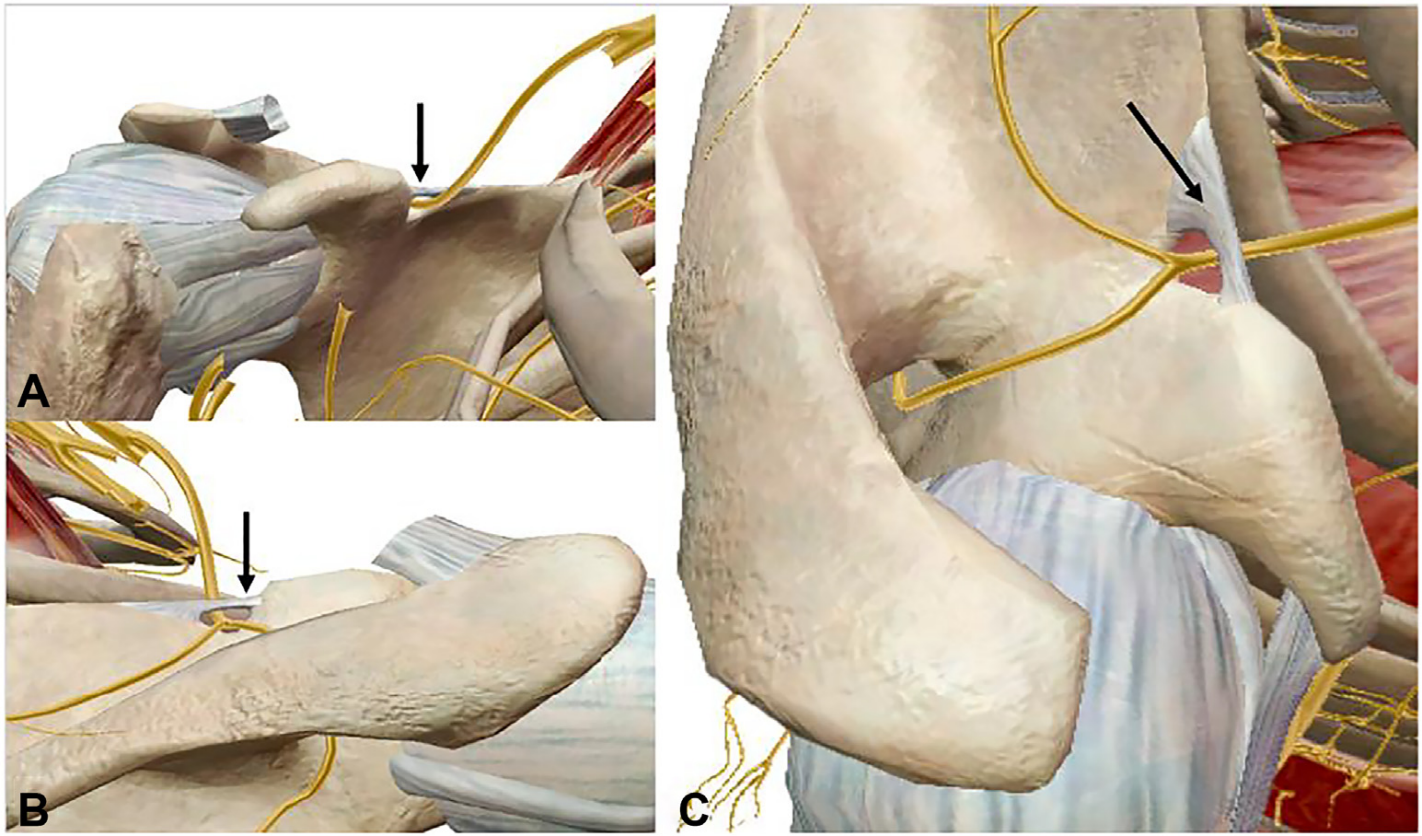
Rendering of the suprascapular nerve and the transverse scapular ligament with subtraction of the deltoid, trapezius, rotator cuff muscles, brachial plexus, vasculature, and PM. **A** Anterior view. **B** Posterior view. **C** Superior view. Note how the branch innervating the supraspinatus takes a sharp turn medially in the supraspinatus fossa immediately beyond the transverse scapular ligament (black arrow indicates the transverse scapular ligament).

An additional pathology simultaneously occurring from this cascade is suprascapular nerve (SSN) entrapment and resultant traction injury at the suprascapular notch (Fig. 5).^66^^,^^67^ Scapular protraction creates chronic stretch injury of the SSN, with symptoms of posterosuperior shoulder pain and radiating pain at the medial border of the scapula exacerbated by overhead activity and even atrophy of the supraspinatus and infraspinatus if left untreated.^68^

## Diagnosis and Indications

Diagnosing NTOS is challenging, with patients experiencing chronic symptoms and often undergoing several surgeries, such as open brachial plexus dissection, scalenectomy, and first rib resection, with variable outcomes because of initial misdiagnosis.^42^^,^^69^ Physicians must first distinguish between NTOS and VTOS. Based on thoracic outlet anatomy (Figure 1, Figure 2, Figure 3) and scapulothoracic biomechanics as presented, the authors contend that PMS creating compression in the retropectoralis minor space is the prime cause of NTOS. Similarly, compression in the supraclavicular thoracic outlet at the scalene triangle and/or costoclavicular space is theorized to cause VTOS. Naturally, there are variations or dual sites of compression that can obfuscate diagnoses; however, it is by this fundamental framework of anatomy that we recommend first honing one’s differential in patients with suspected TOS. The symptoms and signs of NTOS are separated into 4 stages (Table 2). Progressive PM contracture and scapulothoracic motion aberrations lead to worsening symptoms and activity limitations.

**Table 2:** Stages of PMS and Neurogenic Thoracic Outlet

Stage	Symptoms	Clinical Signs	Sport/Activity Participation
1	Mild anterior shoulder, upper chest, trapezial pain	Subtle scapular dyskinesia and protraction	Able to participate
2	Moderate-to-severe pain, with additional radiation around the shoulder and upper arm	Localized tenderness +/− Tinel sign over coracoid Noticeable scapular dyskinesia compared to contralateral side	Hiatus from sport
3	Severe diffuse shoulder pain Significant posterior radiation as suprascapular nerve involvement worsens Worsening periscapular pain	Severe tenderness and Tinel sign over coracoid Marked scapular dyskinesia with limited arm elevation Tenderness at medial scapula (scapulothoracic bursitis) Pain limited and/or objective weakness of supra/infraspinatus	Completely ceased
4	Stage 3, plus additional pain over supraclavicular fossa and neck	Stage 3, plus additional tenderness and Tinel sign over supraclavicular fossa	Completely ceased

### Presentation of vascular TOS

In contrast to PMS leading to NTOS, patients experiencing VTOS present with different symptoms and sites of pain (Table 1). As the scalene contraction draws the first rib superiorly, compression is placed on the subclavian vein in the costoclavicular space and the subclavian artery in the scalene triangle (Fig. 2).^70^ Because the subclavian vein is anterior to the subclavian artery (Figure 1, Figure 2) and dynamic compression in the costoclavicular space primarily occurs anteriorly between the clavicle and first rib, it is no surprise that venous VTOS is more common than arterial VTOS.^71^^,^^72^ Patients note cold sensation in their distal extremity, exhibit venous distention or limb swelling, and unilateral Raynaud-type signs, with advanced cases demonstrating digit ulceration or tissue loss.^73^ Vigilance is required in muscular, young athletes presenting with unilateral pain, swelling, and upper extremity thrombosis, concerning for Paget-Schroetter syndrome, a condition of subclavian vein compression in the costoclavicular space leading to repetitive thrombosis.^74^ The presence of cervical radiculopathy must be evaluated, as the presentation of pain, paresthesia, and weakness in radiculopathy differs from NTOS.^75^

### Diagnosing PMS and NTOS

**Figure 6:**
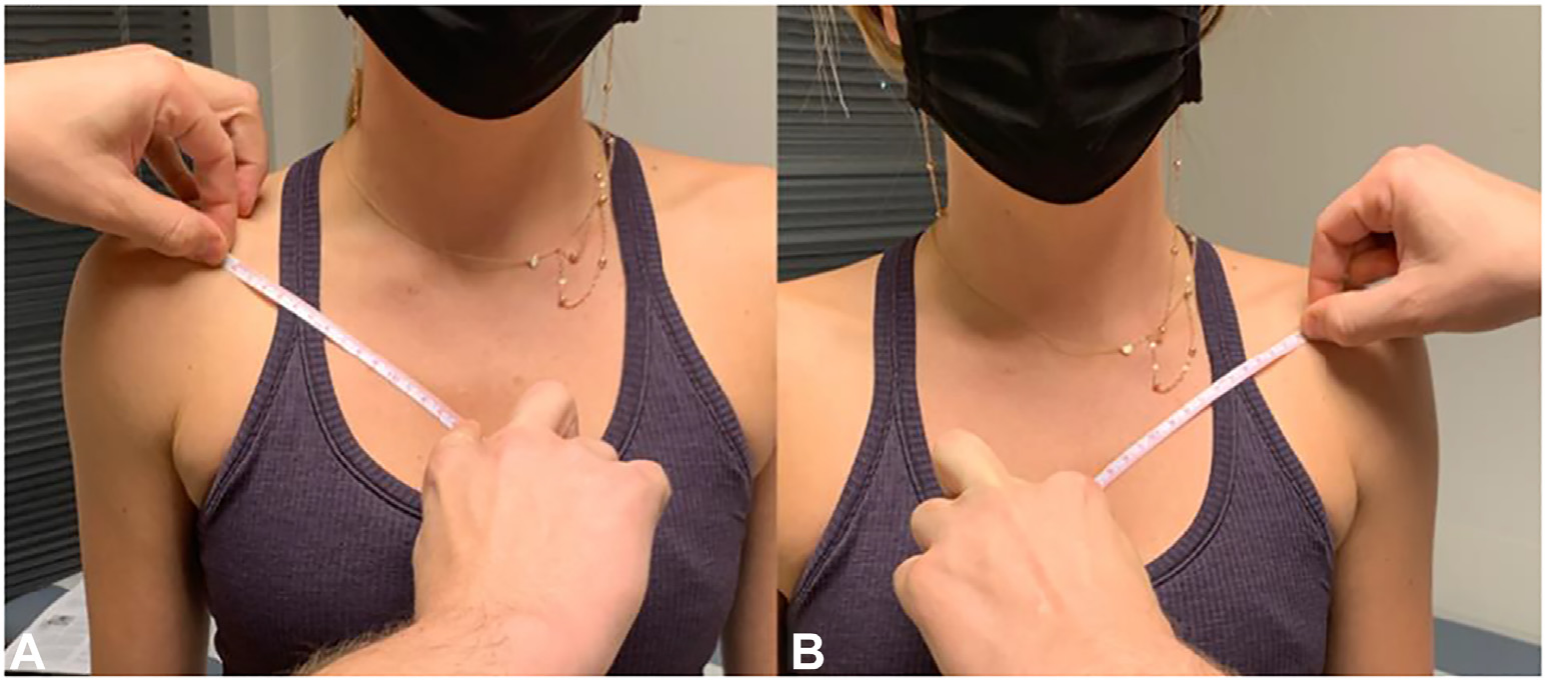
**A** PM length. Measurement of the distance from the medial aspect of the coracoid to the inferior margin of the fourth rib at the sternocostal junction with the patient upright. **B** Measurement of the contralateral side performed for comparison.

Patients with PMS/NTOS typically lack positive findings to classic provocative thoracic outlet tests, such as rotational neck maneuvers and Adson, Wright, Roos, and Cyriax tests.^76^, ^77^, ^78^ In fact, these maneuvers were found unreliable, demonstrating high false-positive and false-negative rates.^79^ The most precise physical findings for PMS are tenderness and a positive Tinel sign over the PM insertion at the superomedial coracoid.^65^^,^^80^ Pain and neurologic symptoms are often worsened by the elevated arm stress test, which positions the shoulder in extension and varying positions of abduction to reproduce pain through compression of the brachial plexus between the PM and thoracic wall.^76^ Finally, PM length should be measured (Fig. 6) from the medial aspect of the coracoid to the inferior aspect of the fourth rib at the sternocostal junction and compared with the contralateral.^45^

**Figure 7:**
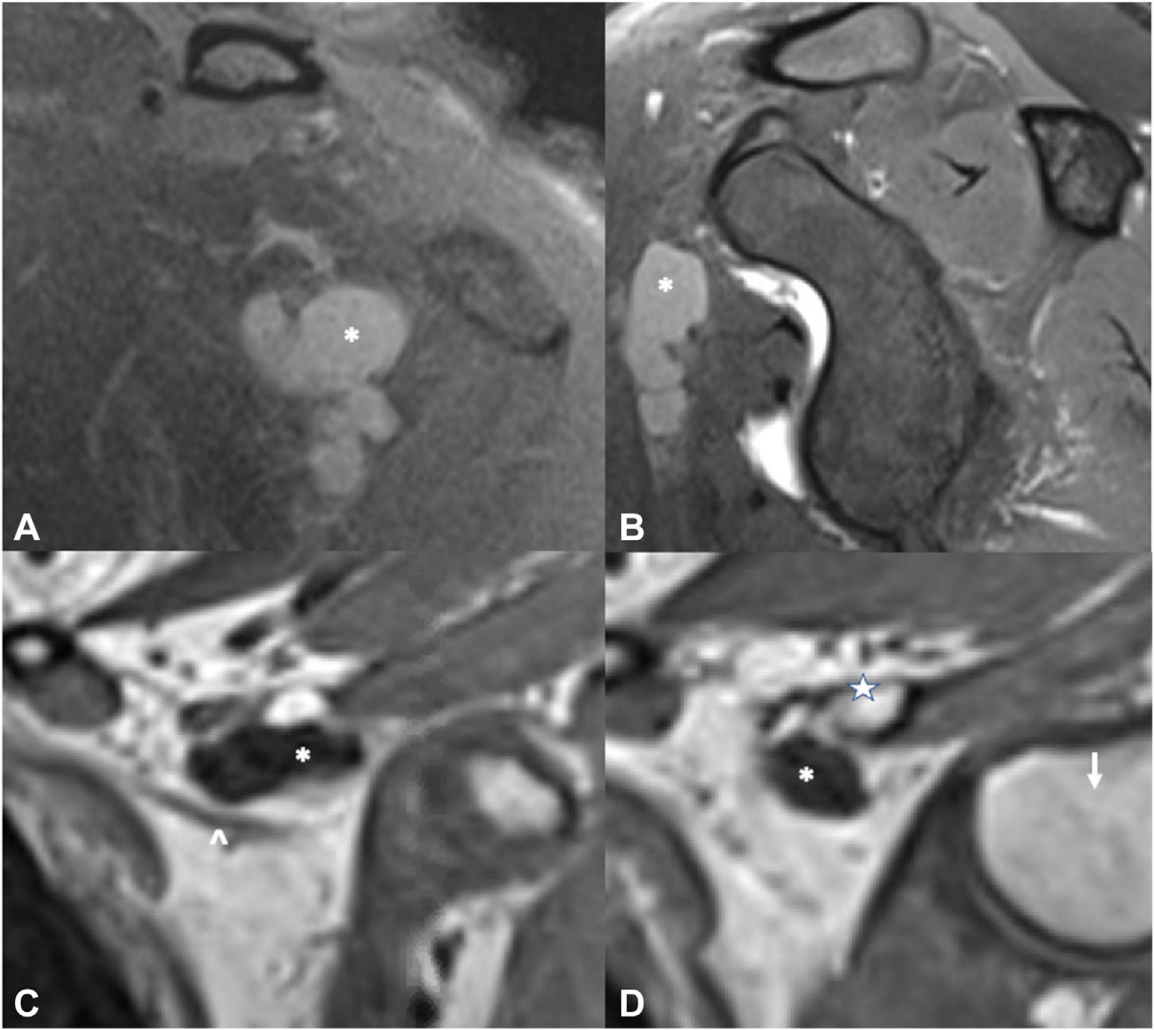
Large subcoracoid cyst noted on the magnetic resonance imaging of the left shoulder and brachial plexus in a patient with vague, deep pain around the anterior shoulder and upper chest, worse with repetitive activity. **A** Coronal view; cyst marked with white ∗. **B** Sagittal view; cyst marked with white ∗. **C** Anterior coronal slice; cyst marked with white ∗, plexus marked with white **ˆ**. **D** Posterior coronal slice; cyst marked with white ∗, coracoid marked with white star, and humeral head marked with white downward arrow. The patient was treated with arthroscopic PM release, cyst decompression, and brachial plexus neurolysis, with resolution of her symptoms and return to activity.

**Figure 8:**
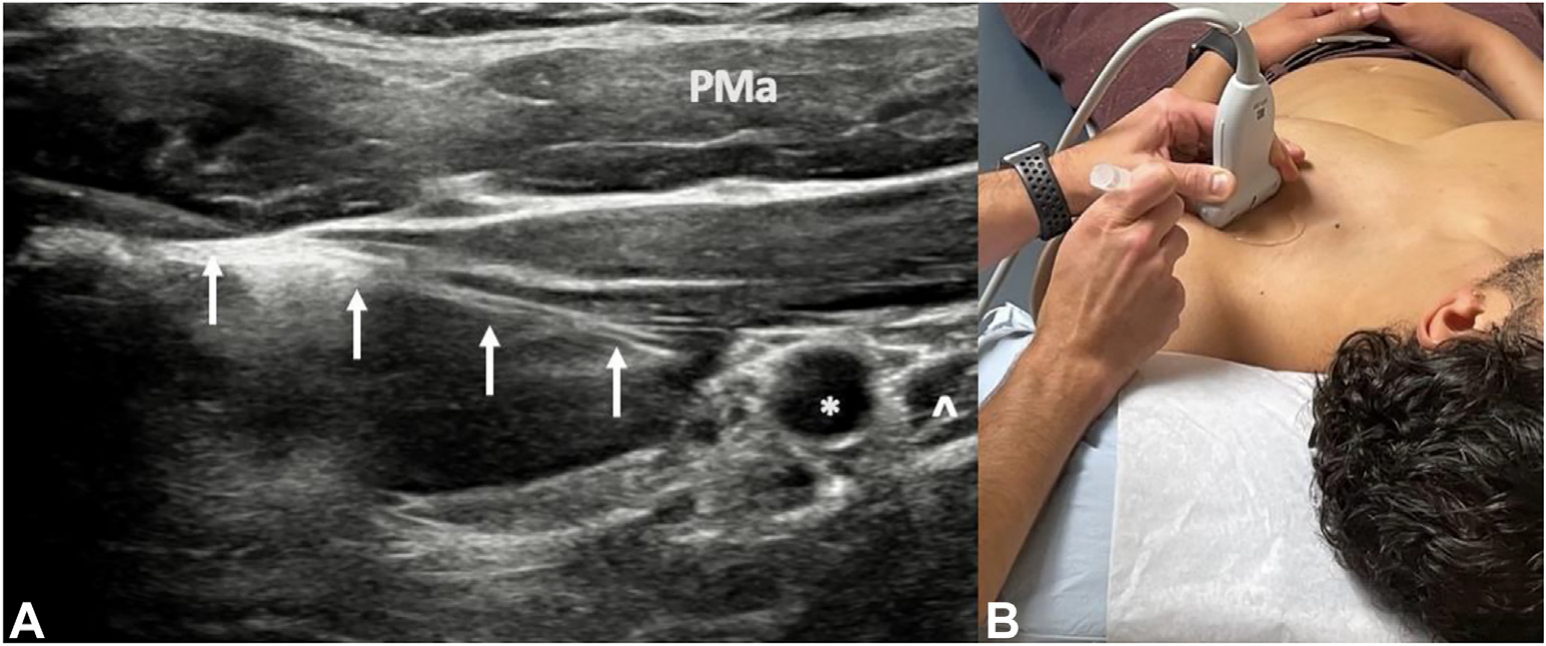
Ultrasound-guided injection of local anesthetic targeted to the PM insertion on the coracoid. The ∗ indicates coracoid, **ˆ** indicates PM insertion, and white upward arrows indicate needle). PMa, pectoralis major.

**Figure 9:**
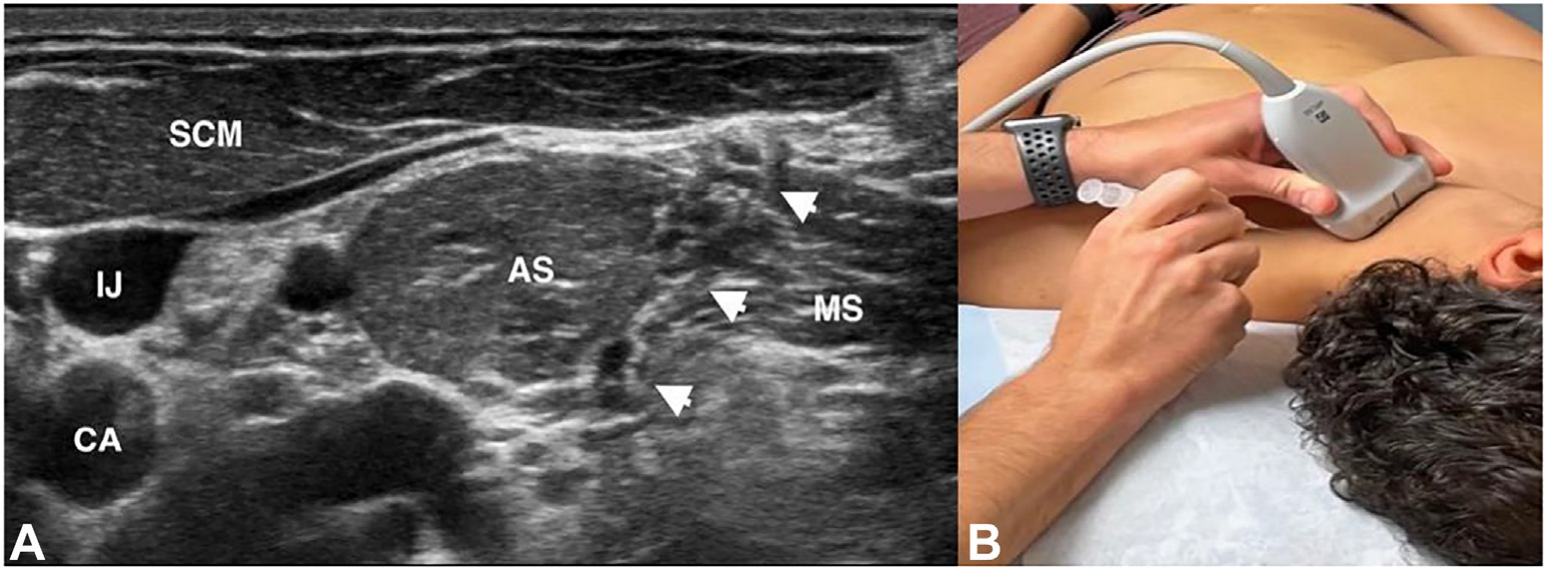
Ultrasound-guided injection of local anesthetic targeted to the scalene triangle. The white arrows indicate the needle. AS, anterior scalene; CA, carotid artery; IJ, internal jugular vein; MS, middle scalene; SCM, sternocleidomastoid.

One must also rule out alternate etiologies such as cervical radiculopathy, anomalous anatomy, VTOS, alternate peripheral neuropathies, and concomitant shoulder pathology. Cervical and shoulder radiographs rule out osseous anomalies such as the cervical ribs, clavicle malunions, or apical lung masses such as Pancoast tumor.^40^^,^^81^^,^^82^ A shoulder magnetic resonance image rules out intra-articular derangements or space-occupying lesions such as subcoracoid cysts (Fig. 7). Electrodiagnostic studies provide insight into possible radiculopathy or alternate peripheral neuropathy. A magnetic resonance imaging of the brachial plexus evaluates possible sites of compression, nerve edema or fibrosis, or pathology along the plexus mimicking NTOS, such as space-occupying lesions or nerve sheath tumors.^83^ Magnetic resonance images are often negative for specific signs of NTOS because of the static nature of the test and lack of obvious compressive lesions.^84^ For patients exhibiting signs of VTOS, one must obtain magnetic resonance or computed tomography angiograms with specific VTOS protocols with dynamic arms elevated/arms down assessment for sites of vascular compression.^85^^,^^86^ Finally, image-guided injection of a local anesthetic targeted to the PM insertion on the coracoid (Fig. 8) is highly beneficial in confirming the diagnosis.^87^^,^^88^ This confirms the diagnosis, and for patients exhibiting a positive injection response, it further correlates with improved outcomes after surgical release.^11^ If symptoms are not improved by injection or if symptoms localize to the supraclavicular area, targeted scalene injections (Fig. 9) can assess potential proximal involvement in advanced stage 4 NTOS.^6^^,^^87^^,^^88^ Guided scalene injections also correlate with a favorable response to surgical intervention.^87^^,^^88^

**Figure 10:**
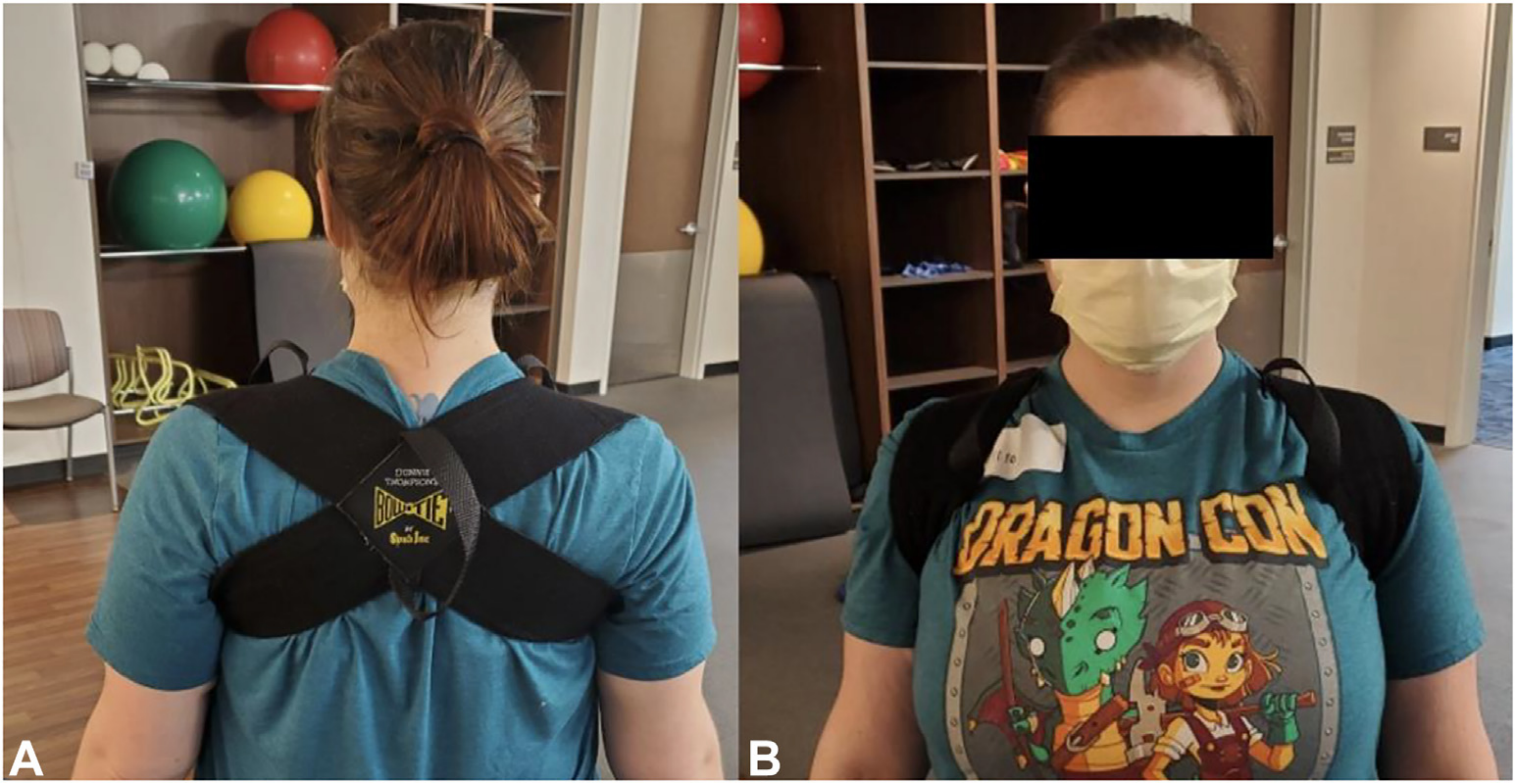
Figure-of-eight brace exerts a constant posterior force on the scapula to combat chronic protraction, augmenting postural retraining.

Initial patient management begins with physical therapy. Emphasis is placed on postural correction, scapular kinematics, and targeted PM stretching. A figure-of-eight brace (Fig. 10) is worn to reposition the scapula in retraction. Patients with continued symptoms despite 6 months of treatment compliance, with the elimination of alternate pathology, and positive injection response (>80% symptom improvement) are offered surgical release.

### Diagnosing suprascapular neuropathy

The diagnosis of concomitant suprascapular nerve compression can be challenging and requires exclusion of alternate diagnoses before surgical intervention.^89^ Patients present with vague posterior shoulder pain, exacerbated by overhead activity. Overt weakness and atrophy are not routinely observed. Tinel sign may be present over the suprascapular notch. The suprascapular nerve stretch test is also performed, with a positive test localizing pain over the posterior shoulder.^68^ Attention must be paid to scapulothoracic motion, as advanced scapular protraction tethers the SSN at the suprascapular notch.^52^ Finally, assess the cervical spine to rule out concomitant radicular symptoms and signs.

**Figure 11:**
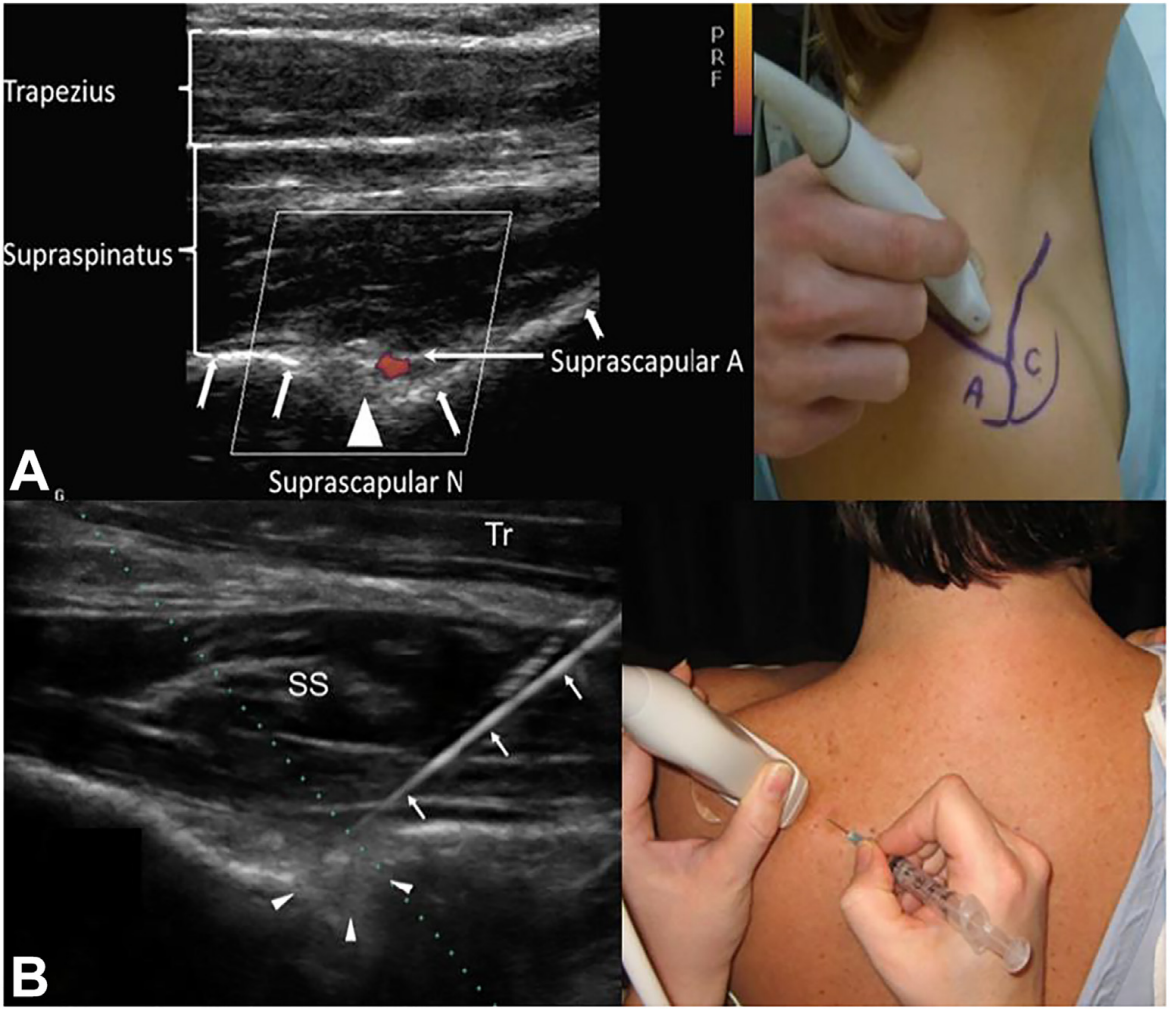
Ultrasound-guided injection of local anesthetic targeted to the suprascapular notch, demonstrating 2 different injection techniques. **A** Superior approach, with Doppler use to identify the suprascapular artery (white arrows with split tails demonstrate needle path and infiltration of anesthetic fluid). **B** Posterior approach. The white arrows indicate the needle, and the white arrowheads indicate the infiltration of fluid in the suprascapular notch. T, trapezius.

Imaging primarily excludes alternate etiologies such as rotator cuff tears, acromioclavicular degeneration, or space-occupying lesions that may compress the SSN.^90^ Similarly, electrodiagnostic studies are not definitive; they assist in ruling out alternate pathologies, such as cervical radiculopathy or brachial plexopathy, but cannot be relied upon to make the diagnosis. Increased supraspinatus muscle insertional activity, fibrillation potentials, positive sharp waves, and decreased motor unit action potentials lend strong evidence for suprascapular neuropathy; however, their absence cannot exclude the diagnosis.^91^ Finally, targeted injections of local anesthetic under image guidance (Fig. 11) provide an excellent assessment of symptoms emanating from the suprascapular notch.^89^^,^^92^^,^^93^

The initial nonsurgical treatment is similar to that of NTOS. Patients who fail 6 months of compliant conservative treatment are considered for surgery. Given the challenges in diagnosis, surgical release is only offered to patients presenting with the above symptoms, lacking alternate etiologies for pain, exhausting nonsurgical treatment, and exhibiting positive injection response (>80% symptom improvement). In our practice, the suprascapular nerve is nearly always concomitantly released during the treatment of NTOS to avoid neglecting any component of the patient’s symptoms.

## Endoscopic Surgical Technique

For patients with persistent symptoms despite scapular-focused and extensive (6 months) nonsurgical treatment, surgical management is warranted. This entails endoscopic PM release, with or without additional SSN release, brachial plexus neurolysis, and subclavius release. The following is a detailed, stepwise description of our surgical technique.

### Patient positioning

The patient is secured and properly padded in the beach chair position. A hydraulic arm holder (Trimano, Arthrex) is often used to manipulate the surgical limb during the procedure. If unavailable, simple traction on the upper limb can also be used. Ensure that the patient is prepared and draped further medially than a typical shoulder arthroscopy. Drape to the sternum medially at the chest and superiorly to the angle of the mandible in the event that supraclavicular access is needed.

### Diagnostic glenohumeral arthroscopy

This is not routinely performed in isolated cases of NTOS. We avoid entering the shoulder joint unless the examination or preoperative imaging detects specific intra-articular pathology that may require intervention. If this is suspected before surgery, a diagnostic shoulder arthroscopy is first performed and any intra-articular pathology is addressed as needed.

### Endoscopic portals

**Figure 12:**
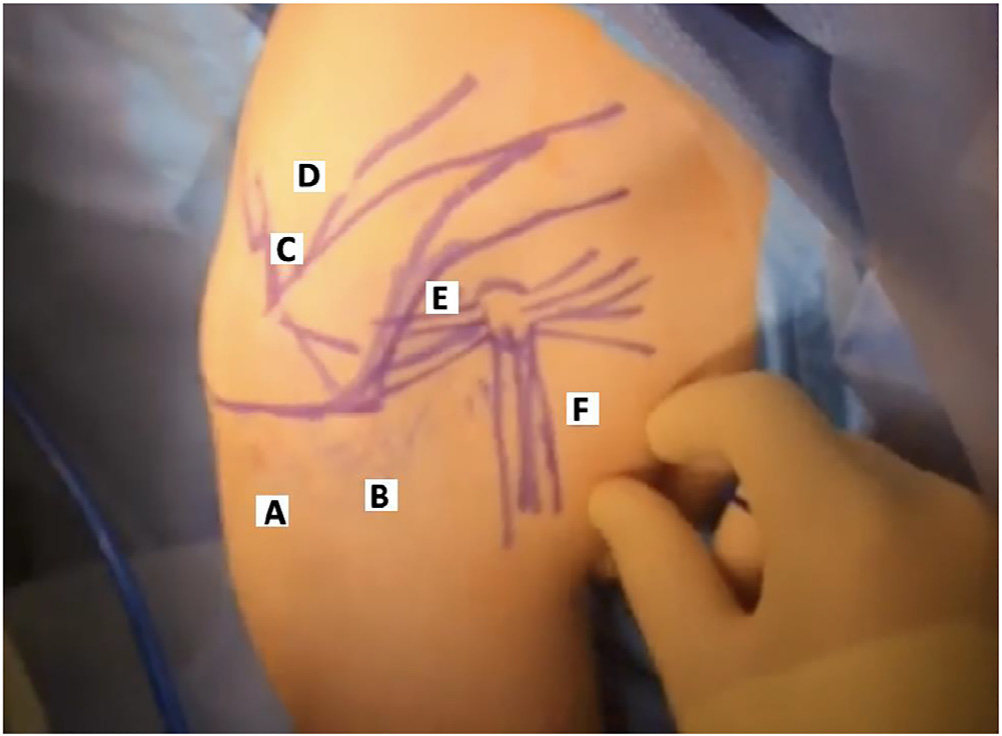
Arthroscopic portals for suprascapular neurolysis, PM release, brachial plexus neurolysis, and infraclavicular thoracic outlet decompression (right shoulder). Portals A–D are used for suprascapular neurolysis. Portals A, B, E, F are used for PM release, brachial plexus neurolysis, and infraclavicular thoracic outlet decompression. A, lateral; B, anterolateral; C, transtrapezial; D, medial transtrapezial; E, anterior; F, anteromedial.

Of note, if concomitant suprascapular neuropathy is present, suprascapular release and neurolysis is performed first, as described by Lafosse et al.^94^ Four portals are used (Fig. 12, portals A–D). Portal A is the lateral subacromial portal, approximately 2–3 cm distal to the palpable lateral acromial border and at the anterior-to-posterior midpoint. This serves as the viewing portal. Portal B is the anterolateral portal, created by needle localization distal to the anterolateral acromial edge and in line with the lateral portal. This serves as the initial working portal for SSN release. Portal C is a transtrapezial portal made with needle localization posterior to the acromioclavicular joint. This is used for retraction of the supraspinatus muscle during SSN release. Portal D is the working portal for transverse scapular ligament release and suprascapular neurolysis and is created with needle localization medial to the prior transtrapezial portal.

Similarly, 4 portals are used for PM release, brachial plexus neurolysis, and infraclavicular thoracic outlet decompression (Fig. 12, portals A, B, E, F). Portals A and B are the same lateral and anterolateral portals for SSN release. These are the initial viewing and working portals, respectively. Portal E is the anterior portal made via needle localization just lateral to the palpable coracoid process. This serves as an additional working and retraction portal within the subdeltoid and subpectoral spaces. Once this is created, the anterolateral portal (B) becomes the viewing portal. Portal F is the anteromedial portal, created with needle localization medial and approximately 2–3 cm inferior to the coracoid. This is the working portal for the actual PM release, brachial plexus neurolysis, and infraclavicular thoracic outlet decompression.

### Suprascapular nerve decompression

**Figure 13:**
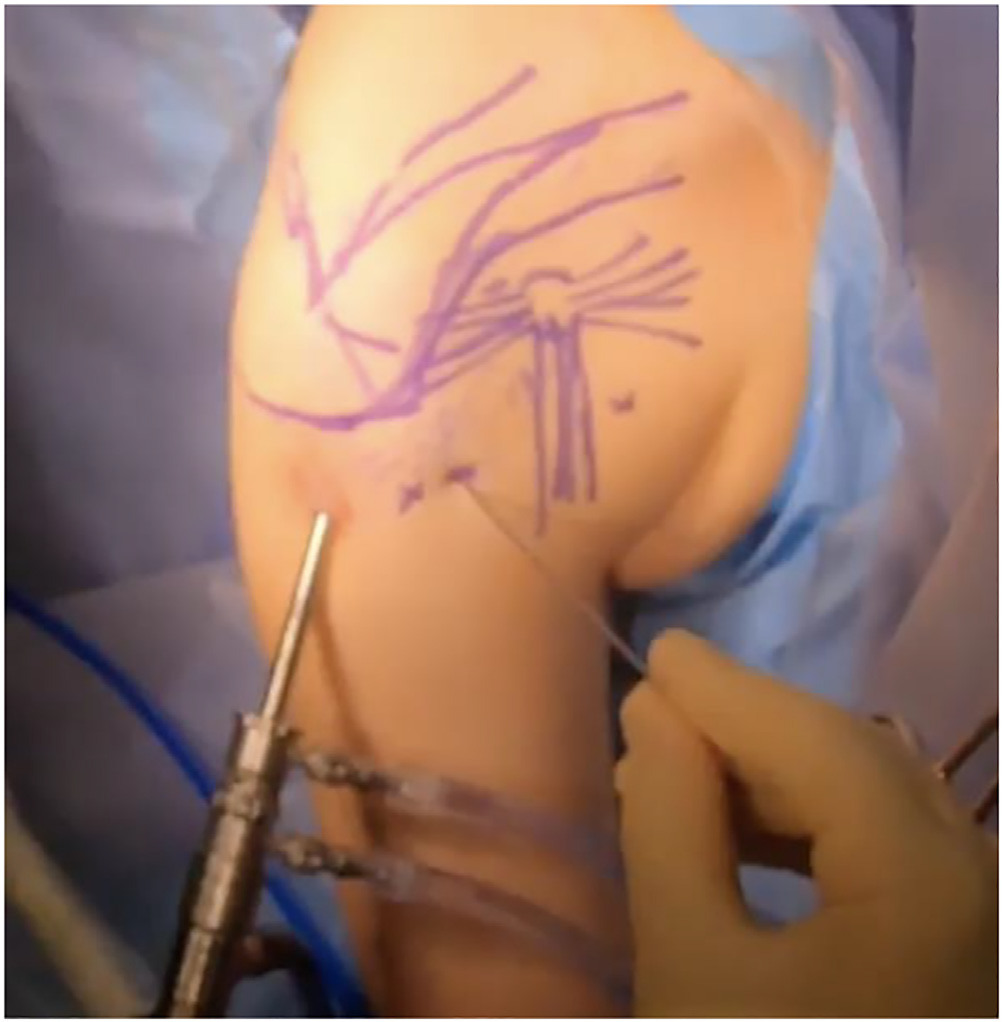
Arthroscope views from the lateral portal while the anterolateral working portal is created via needle localization. These are the initial viewing and working portals for both suprascapular nerve release and PM release.

**Figure 14:**
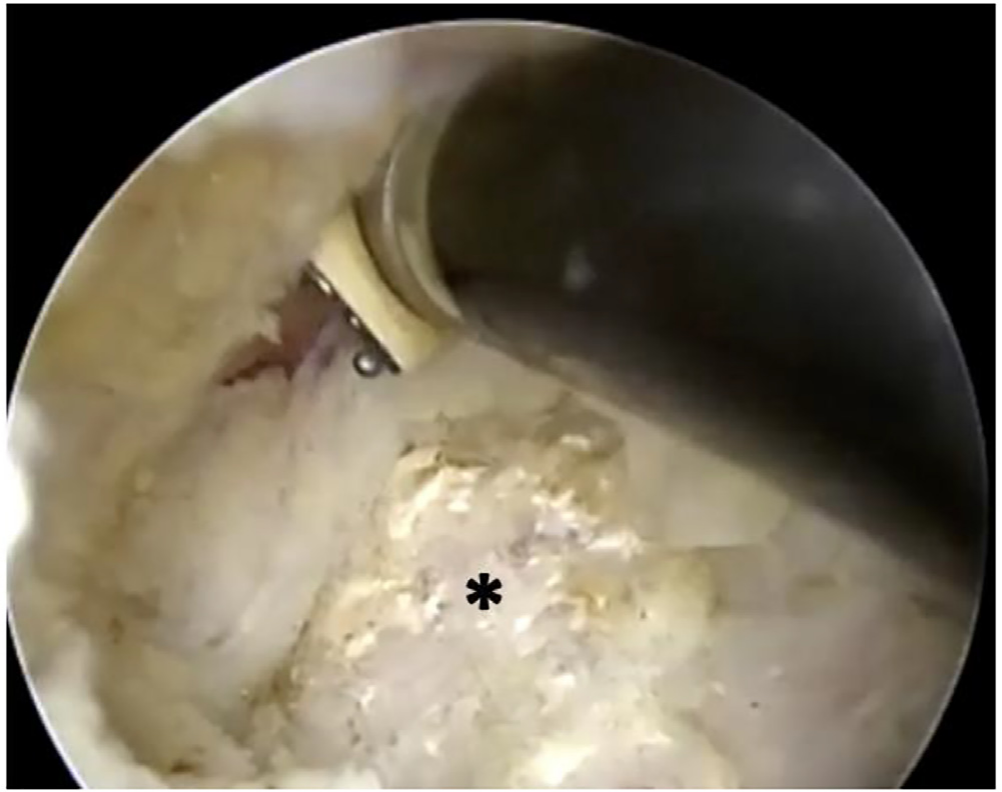
Viewing from the same lateral portal, the base of the coracoid is seen below the electrocautery wand. Medial advancement progresses toward the suprascapular notch. The ∗ indicates the base of the coracoid.

A lateral subacromial portal is first created for viewing, followed by the anterolateral working portal (Fig. 13). Subacromial decompression is performed with a combination of shaver and electrocautery. The coracoacromial (CA) ligament is next identified and serves as the landmark for SSN release. The anterior edge of the supraspinatus (SS) tendon and muscle is identified beneath the CA ligament. The posterior border of the CA ligament is followed medially toward the base of the coracoid (Fig. 14). In doing so, the anterior SS must be gradually released and elevated to permit medial advancement. An electrocautery wand is preferentially used in a pulsing fashion to liberate adhesions around the anterior SS, permitting posterior mobilization.

**Figure 15:**
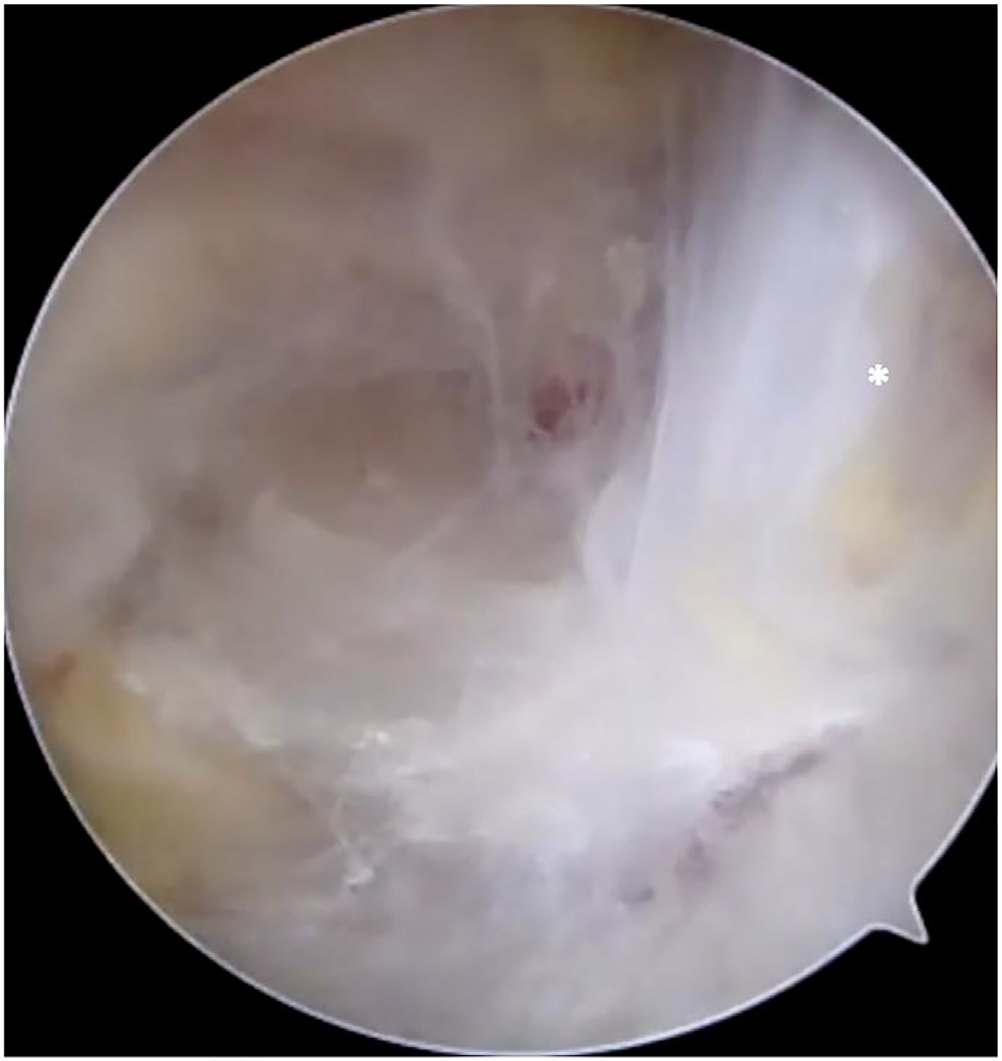
Viewing from the same lateral portal, the vertically oriented fibers of the coracoclavicular ligaments are seen (white ∗) as one advances posteriorly around the coracoid base.

A transtrapezial portal is next made via needle localization. A blunt arthroscopic trocar is introduced through this portal, serving as a retractor to pull the SS posteriorly as one progresses medially. Once the CA ligament is followed to the base of the coracoid, one must remain cognizant of multiple crossing vessels. Electrocautery used in pulsing fashion to clear adhesions around the posterior coracoid pre-empts unnecessary bleeding. At the coracoid base, one can see the vertically oriented coracoclavicular ligaments (Fig. 15). The trocar through the transtrapezial portal is repositioned for SS retraction as needed, and the scope is advanced medially, posterior to the coracoclavicular ligaments and coracoid base.

**Figure 16:**
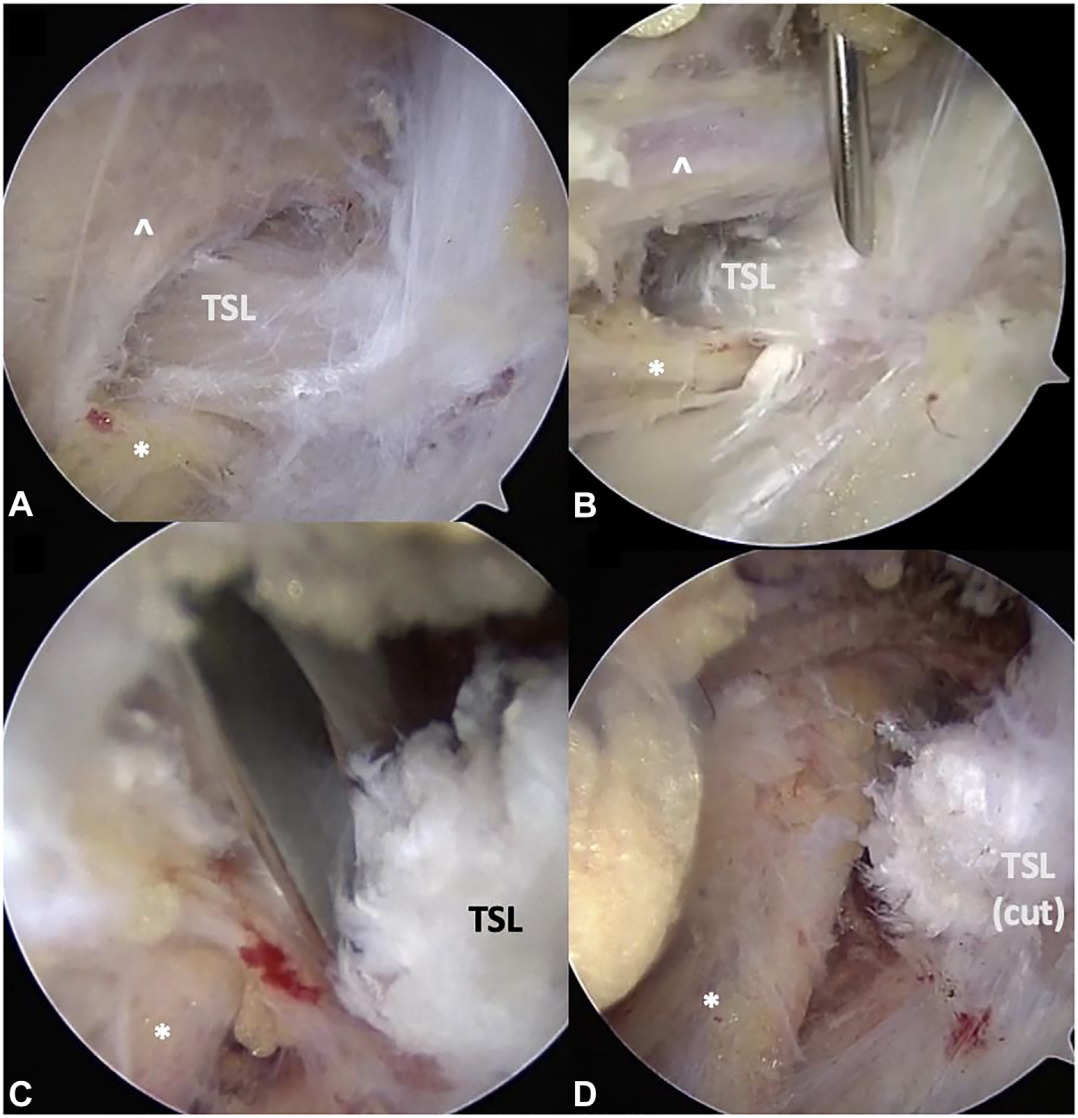
Arthroscopic suprascapular nerve release of the right shoulder. **A** View from the subacromial space via the lateral portal and 30° arthroscope, progressing medially following the CA ligament and releasing along the anterior border of the supraspinatus muscle until the transverse scapular ligament is encountered posterior to the coracoid. **B** Needle localization creating the medial transtrapezial working portal. **C** Arthroscopic scissors introduced through this working portal, releasing the transverse scapular ligament. The suprascapular nerve is safely visualized inferior to the ligament. The suprascapular artery runs anterior to posterior over the ligament, and is displaced posterior-medial to the scissors to ensure it remains protected. **D** Released suprascapular nerve. The ∗ indicates suprascapular nerve, and **ˆ** indicates suprascapular artery. TSL, transverse scapular ligament.

In this location, the suprascapular notch is encountered (Fig. 16). Beforeactually visualizing the transverse scapular ligament, the large suprascapular artery is seen coursing medial to lateral, superficial, and relatively perpendicular to the ligament (Fig. 16A). To release adhesions and mobilize the artery, both the trocar and blunt back end of the electrocautery wand are used in sweeping motions longitudinally along the artery. Once mobilized, the artery is retracted posteriorly to fully expose the transverse scapular ligament. Of note, the presence of a subligamentous suprascapular artery has been described.^95^ Although rare, one must be aware of this anatomic variation if the suprascapular artery is not readily visualized coursing above the ligament. The transverse scapular ligament is seen as thick, white fibers running obliquely in an anterior-to-posterior direction. The suprascapular nerve passes inferior to the ligament in the same medial-to-lateral direction as the overlying artery. It is not uncommon to encounter a supraspinatus motor branch arising from the main suprascapular nerve proximal to the ligament and passing above it. This branch must be respected during the lateral-to-medial progression of neurolysis. Once the ligament is exposed, an additional working portal is created with needle localization (Fig. 16B), medial to the transtrapezial retraction portal. Arthroscopic scissors are introduced through this portal. The transverse scapular ligament is released at the anterior portion, closer to the coracoid base, as this is further from the artery and easier to clearly visualize (Fig. 16C). The ligament is quite thick, and a gnawing motion with the scissors facilitates controlled release. Although rare, it should be noted that ossification of the ligament can occur.^15^ In this scenario, a Kerrison rongeur is used for release.

Following transverse scapular ligament release, the SSN is readily apparent. Neurolysis is performed gently using arthroscopic scissors, spreading longitudinally on either side of the nerve. The blunt trocar is similarly used to release loose areolar tissue and adhesions along the nerve. Adequate neurolysis is confirmed when the SSN is freely mobile within the suprascapular notch (Fig. 16D).

### PM release

**Figure 17:**
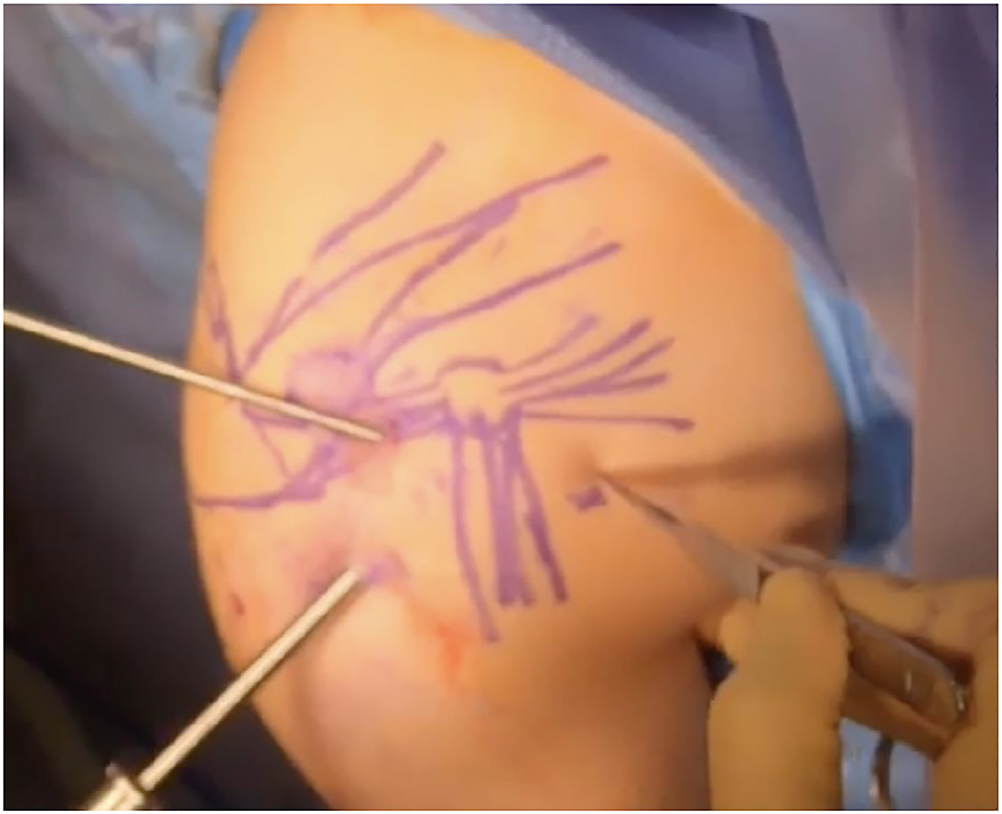
Arthroscope now transitioned to the anterolateral portal for viewing. The anterior portal was also created, with a switching stick serving as a retractor in the subdeltoid and subpectoral space. Scalpel is preparing to create the anteromedial working portal following needle localization.

In patients with concomitant suprascapular neuropathy and NTOS, as is often the case, attention is then turned to the PM. The lateral portal is initially used for viewing, and the anterolateral portal serves as the preliminary working portal (Figure 12, Figure 13). If not already performed (cases without concomitant suprascapular neuropathy), the subacromial space is decompressed until the CA ligament is identified. The subdeltoid space is then cleared of adhesions and areolar tissue. Avoid violation of the deltoid fascia, as this hastens fluid extravasation and development of subcutaneous edema. Decompressing the subdeltoid space permits easy maneuverability of the scope and instruments to the anterior aspect of the CA ligament and eventually the anterior coracoid. After subdeltoid space decompression, an anterior portal is made just lateral to the palpable coracoid with needle localization. This allows retraction within the subdeltoid and subpectoral space and further serves as an additional working portal. The scope is then transitioned from the lateral portal to the anterolateral portal for viewing (Fig. 17).

**Figure 18:**
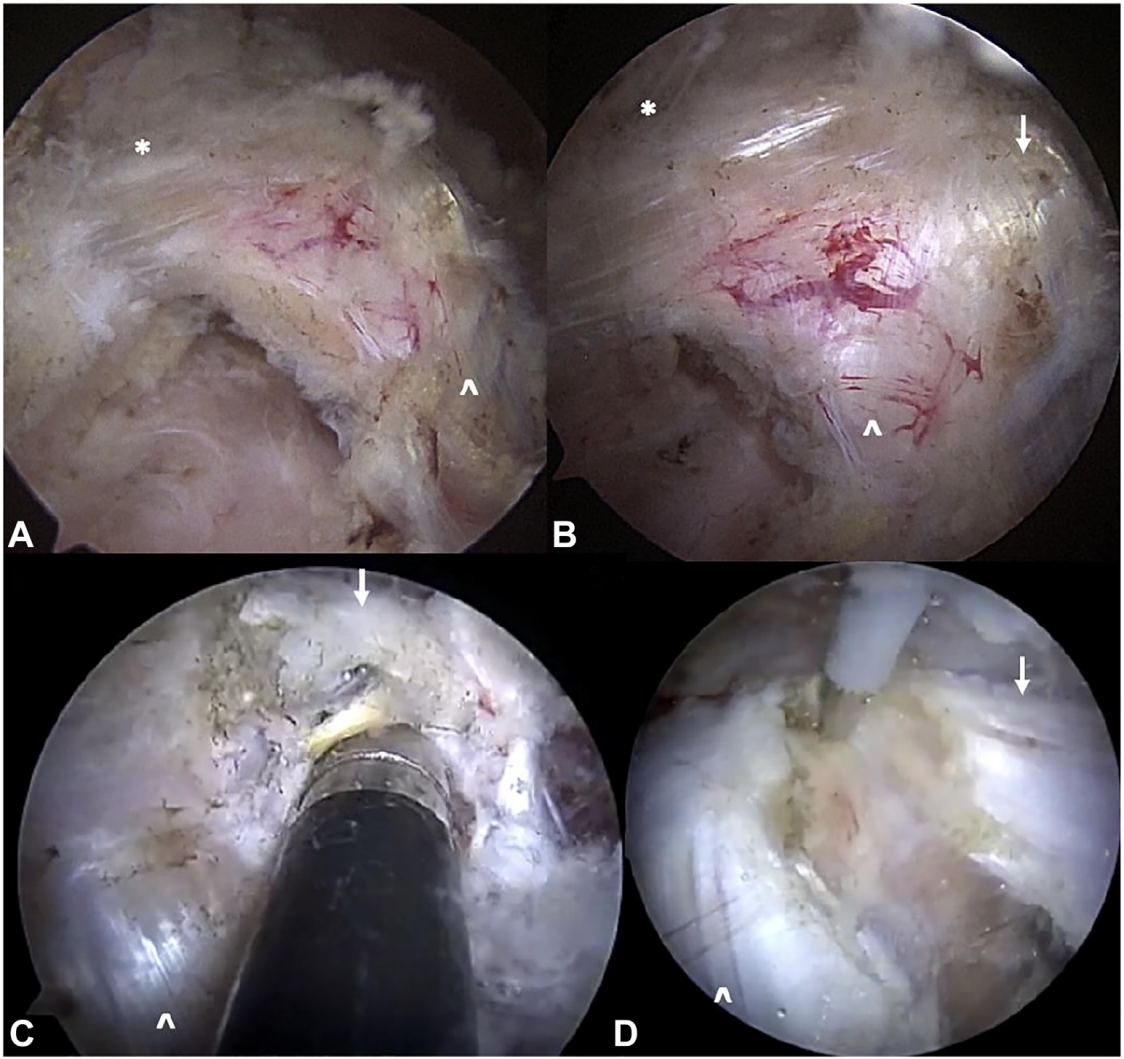
Arthroscopic PM release of the right shoulder. **A** View from the anterolateral portal with standard 30° arthroscope, demonstrating the coracoacromial ligament (∗) and conjoint tendon (**ˆ**). **B** View from the same anterolateral portal with 70° arthroscope, demonstrating the classic “T” appearance of the coracoacromial ligament (∗), conjoint tendon (**ˆ**), and PM (downward white arrow) converging on the coracoid process. **C** Release of the PM tendon insertion (downward white arrow) off the medial coracoid using electrocautery. The conjoint tendon is also seen in this view (**ˆ**). **D** Continued release of the coracoid with inferior and medial retraction of the PM tendon (downward white arrow). This retraction of the released tendon is routinely noted in patients with PMS and NTOS.

Through the anterolateral portal, the scope is positioned anterior and lateral to the coracoid within the subdeltoid space. The viewing angle is adjusted to look directly toward the coracoid (Fig. 18A). Any remaining adhesions and areolar tissue overlying the coracoid are cleared. A combination of shaver and electrocautery is typically used to clear the tissue and minimize bleeding over the superior aspect of the coracoid. The release is sufficient when one can clearly see the conjoint tendon originating from the coracoid and continuing distally and the CA ligament coursing laterally (Fig. 18A). The PM tendon insertion on the medial coracoid cannot be visualized adequately for safe release.

The anteromedial portal is next created with needle localization, serving as the working portal for PM release. The arthroscope is switched to a 70° viewing angle but is maintained in the anterolateral portal. This change in angle affords an excellent en face view directly at the coracoid. If a 70° scope is unavailable, adequate visualization is still possible with a 30° scope and additional anterior portal(s) to manipulate the viewing angle to achieve the en face view. The coracoacromial ligament, conjoint tendon, and PM tendon create a “T” shape, converging on the coracoid process (Fig. 18B). Electrocautery and shaver are used to clear any remaining adhesions around the superior aspect of the coracoid and overlying the PM tendon. Using electrocautery, a plane is created between the conjoint tendon laterally and PM tendon medially. This is performed in sweeping motions from inferior to superior. Care is taken not to plunge deep, as the brachial plexus cords and axillary vessels lie within this retropectoralis minor space. The extent of adhesions between the 2 tendons can be quite variable; in some, this plane is easily and quickly developed, whereas others have thick interconnections that must be released. Regardless, developing this interval is imperative to adequately visualize the insertion of the PM tendon on the medial coracoid and to identify and protect the underlying brachial plexus.

**Figure 19:**
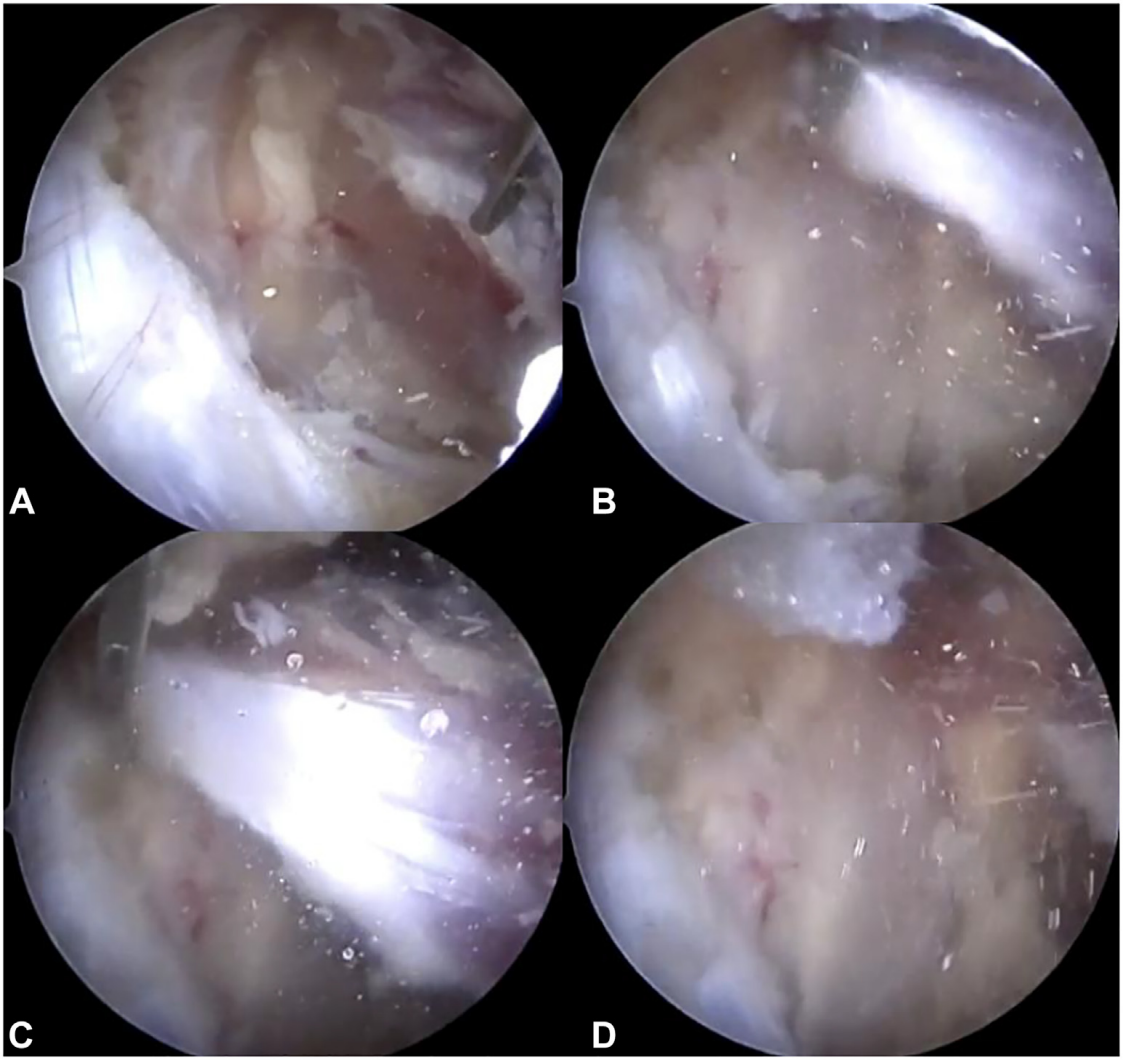
Grasper maneuver to appreciate PM tension after release. **A** The grasper is used to hold the released lateral edge of the tendon (right side of image). **B, C** The grasper pulls the tendon laterally, back toward the coracoid insertion. **D** Immediate inferomedial tendon retraction occurs upon release of the grasper.

Electrocautery is then used to release the PM tendon insertion directly off the medial coracoid, beginning at the inferior aspect of the insertion and continuing superiorly (Fig. 18C). Release is facilitated by initially facing the ablating surface directly toward the medial coracoid bone and pulsing the current as one’s forearm is rotated to turn the ablating surface superiorly into the tendon fibers. This permits a more controlled release than simply pushing or pulling with the electrocautery, given the close proximity of critical neurovascular structures. Often, additional adhesions are present after releasing the PM tendon, most pronounced superiorly and just deep to the tendon insertion. Remaining adhesions are released until the loose fatty tissue is seen deep to the level of the tendon (Fig. 18D). Complete release is confirmed when the tendon retracts medially and inferiorly (Fig. 19). To appreciate the amount of tension and contracture of the PM in these cases, the following maneuver can be performed: using a grasper (Fig. 19A), pull the released edge of the PM tendon superiorly and laterally, back toward the medial coracoid (Fig. 19B, C). Upon releasing the grasper, immediate recoil of the PM occurs in an inferomedial direction, toward its origin on the anterior ribs (Fig. 19D).

### Brachial plexus neurolysis and infraclavicular thoracic outlet decompression

**Figure 20:**
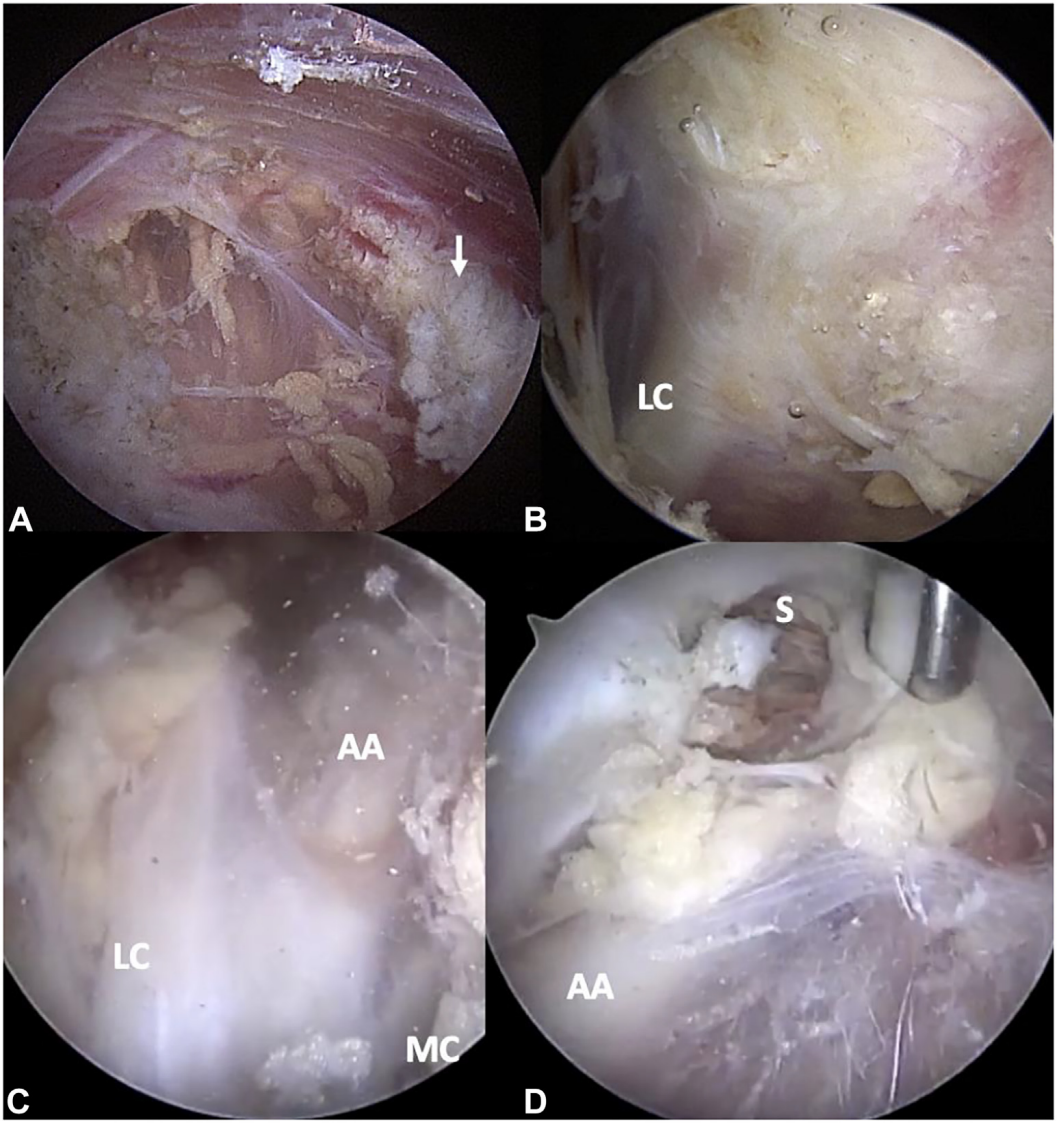
Brachial plexus arthroscopic neurolysis following PM release. **A** PM completely released (downward white arrow) with classic inferomedial retraction. Loose areolar tissue is seen deep, indicating that the retropectoralis minor space is now open. **B** Release of adhesions and areolar tissue in the retropectoralis minor space uncovers the lateral cord of the brachial plexus. **C** Continued proximal release uncovers the axillary artery. The medial cord is partially visualized at the bottom right. **D** Further proximal release presents the subclavius muscle on the inferior surface of the clavicle. This is released with electrocautery, completing the infraclavicular thoracic outlet release. AA, axillary artery; LC, lateral cord; MC, medial cord; S, subclavius muscle).

At this stage, suprascapular neurolysis and PM tenotomy are complete (Fig. 20A). If the patient has preoperative evidence of additional proximal neural compression, arthroscopic brachial plexus neurolysis is performed. The viewing and working portals are not changed; 70° arthroscope in the anterolateral portal and the anteromedial portal remains the working portal. The debridement of loose fatty tissue just deep to the level of the PM tendon is begun with a shaver. Face the cutting surface toward the arthroscope, as the brachial plexus cords and axillary vessels are within this fatty tissue. As the tissue is cleared, the neurovascular structures come into view, coursing obliquely in a proximal-medial to distal-lateral direction (Fig. 20B, C). Often the first tell-tale sign is visualization of small, transverse blood vessels coursing as part of the intraneural vasculature within the brachial plexus cords. Once these are seen, the shaver is removed, and an electrocautery wand is inserted through the same anteromedial working portal. The remaining adhesions around the plexus are released with the wand, pulsing the coagulation current and using long sweeping motions parallel to the longitudinal course of the neurovascular structures. The cauterizing surface of the wand is oriented toward the endoscope rather than the tissue itself. In this manner, adhesions and areolar tissue around the plexus are teased away gradually and safely. Fibrous bands are encountered, particularly as one progresses further proximally closer to the clavicle. These can be fairly robust, often running in a transverse direction. Given the transverse orientation, they are safe to release using controlled ablation. Once the release of the deep fatty and areolar tissue is complete, one can easily appreciate the rhythmic pulsations of the axillary artery and see the surrounding lateral and medial cords of the brachial plexus (Fig. 20C). The posterior cord is deep into the axillary artery and is rarely seen, as retracting axillary vessels in this location is unnecessary.

Upon the visualization of neurovascular structures, attention is turned to release of the subclavius muscle from the inferior surface of the clavicle. Again, viewing and working portals are unchanged, with the 70° arthroscope viewing angle maneuvered to look proximally. The now-released brachial plexus cords and axillary vessels are followed proximally and medially until the subclavius muscle is encountered at the inferior surface of the clavicle. The muscle fibers are readily distinct from the surrounding tissue, coursing in a medial-to-lateral direction. Additional fascial bands along this course are released as previously described. The release of the subclavius muscle is performed directly through the visible muscle belly, using an electrocautery wand to minimize bleeding (Fig. 20D). The muscle is fully released in this fashion until the bony undersurface of the clavicle is encountered. At this stage, the entire infraclavicular thoracic outlet has been decompressed. Arthroscopic portals are closed in standard fashion, and a soft dressing and simple sling are applied.

The postoperative protocol is provided in Table 3. The early range of motion and targeted stretching begin under therapist guidance, along with figure-of-eight bracing. These protocols involve comprehensive PM stretching, postural retraining, and scapulohumeral rhythm retraining.^48^^,^^63^^,^^96^, ^97^, ^98^ Aggressive periscapular muscle strengthening is initiated between 4 and 6 weeks, with most patients returning to overhead activity and sports by 4 months after surgery. During rehabilitation, strict attention to the core, hip, and lower extremity strengthening and coordination is emphasized.^98^^,^^99^

**Table 3:** Postoperative Protocol After Arthroscopic NTOS Release

Time Point	Phase	Details
Wks 0–2	Phase 1: immobilization	- Simple sling immediately after surgery - Transition to figure-of-eight brace at the first postoperative visit - Passive and active elbow, wrist, and hand motion
Wks 2–6	Phase 2: range of motion and scapula retraining	- Progress from passive-to-active–assisted to active shoulder motion - Periscapular strengthening, retrain scapular kinematics, and PM stretching - Continue using the figure-of-eight brace - Pool therapy encouraged
Wks 6–12	Phase 3: strengthening	- Full active/passive shoulder motion - More aggressive strengthening with a progression to eccentric strengthening - Continue postural retraining and scapulohumeral rhythm kinematics, continue the use of figure-of-eight brace
Wks 12–16	Phase 4: sports and activity specific	- Continue phase 3 therapy - Wean the use of figure-of-eight brace - Gradual return to sport and activity

In conclusion, NTOS is challenging to recognize, diagnose, and treat. Sound knowledge of scapulothoracic biomechanics and thoracic outlet anatomy is essential to distinguish NTOS from VTOS. The diagnostic algorithm must rule out various mimicking etiologies. Ultrasound-guided injections are a mainstay of diagnosis and are recommended before invasive treatment as they are both diagnostic and prognostic. Therapy to correct scapular dyskinesia and stretching of the PM is often successful. Refractory cases are treated with endoscopic management, avoiding the morbidity of traditional open procedures. Releasing the PM yields high rates of success and can be combined with suprascapular nerve decompression, brachial plexus neurolysis, and subclavius release, as dictated by the preoperative work-up.
